# Rapid hydrothermal triggering of induced seismicity at the Coso geothermal field

**DOI:** 10.1038/s41598-026-38146-x

**Published:** 2026-02-03

**Authors:** Joanna M. Holmgren, J. Ole Kaven, Volker Oye

**Affiliations:** 1 NORSAR, Gunnar Randers vei 15, Kjeller, N-2007 Norway; 2 Earthquake Science Center, U.S. Geological Survey, Pasadena, CA USA

**Keywords:** Natural hazards, Solid Earth sciences

## Abstract

The long-term producing Coso Geothermal Field (CGF) in California operates over 100 wells tapping into a reservoir characterized by an extensive fracture network, complex fluid pathways, and regular seismic activity. Understanding the interaction between seismicity and injection can shed important light on the hydrothermal characteristics of the field. Here, we analyze 15 years of local seismic and daily operational data from the CGF, identifying a strong correlation between short-term increase in seismicity rate and seasonal volumetric and temperature variations in the reinjected fluid. Furthermore, the seismic footprint during peak injection of colder fluids reveals a near-instantaneous response up to 2 km away from the injection well, too rapid for pore pressure diffusion alone. This short-term and distant response is observed to have directional preference, indicating structural or permeability anisotropy within the reservoir. Additionally, the seismic response correlates with the initial volumetric increase of colder fluids, but also with temperature decrease during stable injection periods, suggesting thermal effects alone can play an important role in triggering distant seismicity.

**Supplementary Information:**

The online version contains supplementary material available at 10.1038/s41598-026-38146-x.

## Introduction

Several underlying mechanisms drive induced seismicity in long-term producing, conventional geothermal fields, with the dominant ones including changes in pore pressure as well as poro- and thermoelastic stress transfer^1–4^. In general, the long-term trends, such as reservoir subsidence, have been found to be driven primarily by thermal effects^3,5–10^, whereas short-term trends of increased seismicity are driven by pressure effects^7,9–12^. When considering the spatial evolution of seismicity, cooling and thermal contraction is believed to induce seismicity mainly near the injection wells, caused by the temperature contrast between the injected fluid and geothermal reservoir^2,13^. Because of the slower process of heat transfer, injection-induced seismicity at larger distances is commonly linked to pore pressure diffusion^11^ or poroelastic stresses^14,15^, although thermoelastic stresses have also been found to transmit beyond the cooled region^16^. Considering the complexity of geothermal reservoirs and how they respond to long-term production, more case studies documenting trends and correlations between operational parameters and the resultant induced seismicity can help further our understanding of the spatiotemporal evolution of these underlying mechanisms, which we aim to do here using the Coso Geothermal Field (CGF) in California.

**Fig. 1:**
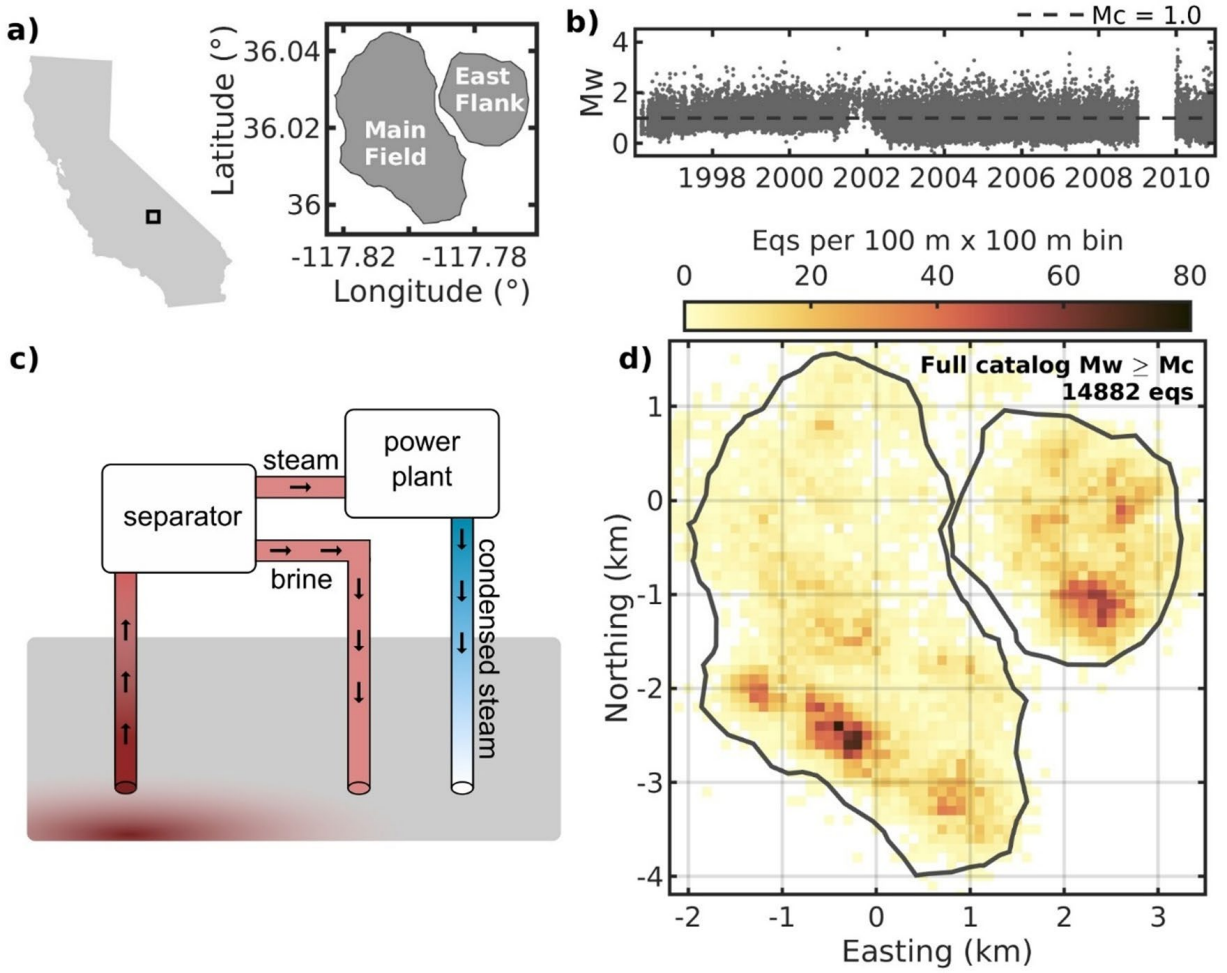
(**a**) Map of the Coso Geothermal Field (CGF) study area and its two subfields Main Field and East Flank. (**b**) Moment magnitude ($\:{M}_{w}$) plotted against origin time for the full catalog. (**c**) Simplified overview of the two types of reinjection fluids at a flash steam plant. In practice, these output fluids are mixed to varying degrees during reinjection. (**d**) Spatial density map of $\:{M}_{w}$ ≥ 1.0 seismicity for the full catalog. Maps are created using MATLAB^33^ (version R2024b, https://www.mathworks.com), with the California border extracted from^34^ and the subfield outlines extracted by clustering the earthquake catalog using Density-Based Spatial Clustering of Applications with Noise (DBSCAN)^35^ (epsilon neighborhood = 260 m and minimum neighbors = 45).

The CGF reservoir is located within the tectonically active Coso Range in the eastern California shear zone^17,18^ (Fig. 1a) and is characterized by an extensive fracture network overlying a heat source at ≥ 5–6 km depth^19–21^. With production starting in 1987, long-term operations have led to an increase in seismicity primarily driven by thermo-hydro-mechanical (THM) coupling processes^22,23^, with seismicity generally clustering near injection wells^23^. For shorter timescales, Holmgren et al.^12^ found that operational pauses at the CGF, i.e., shut ins, trigger seismicity near production wells. This phenomenon has been observed at several geothermal fields and is due to the pressure spike observed when the flow of geothermal fluids is temporarily stopped^12,24,25^. At the CGF, shut ins are typically carried out in the spring for maintenance and last between 2 and 10 days. Additional short-term CGF seismicity trends have been linked to solid-earth tides^26^ and dynamic triggering from distant earthquakes^26–28^.

For geothermal production, the CGF runs flash steam power plants^29^ in which hot geothermal fluid (brine) is extracted and sent to a separator, which separates the steam from the brine to be sent to the turbines for power generation (Fig. 1c). Simplified, this results in two end-member reinjection fluids (i.e., they can be mixed before injection): the left-over and still-hot brine from the separator and the cooler condensed steam output from the power plants. Being located in California, peak production demand at the CGF occurred during summer months for our study period 1996–2010. However, the higher evaporation rates in the summer also leads to less fluid to reinject^30^. This is caused by less condensate from the cooling towers due to evaporation, and because the power plants’ condensed steam output is partly stored uncovered at the surface before reinjection and also more susceptible to evaporation.

While it has been shown that the CGF long-term production has led to an increased background seismicity rate compared to pre-production^31^, previous studies relying on monthly hydraulic operational data and a regional earthquake catalog have reported both limited^26,31^ and significant^32^ correlation between the seismicity and operational activity. Here, we use daily operational data and a local earthquake catalog to investigate spatiotemporal trends and identify seasonality at the CGF. We find annual patterns and clustering in seismicity vary spatially throughout the field, strongly linked to the volume and temperature of the reinjected fluid. Moreover, we observe far-reaching (~ 2 km) near-instantaneous seismic response with clear directional preference (north), which leads us to suggest permeability anisotropy and/or fracture-dominated thermo-poro-elastic effects within specific regions.

## Data

We use the 1996–2010 earthquake catalog recorded by the U.S. Navy Geothermal Program Office local seismic network (Fig. 1b and d). The catalog consists of 63,865 earthquakes from the CGF, mainly from its two subfields Main Field and East Flank (Fig. 1a), located by Kaven et al.^36^, with average horizontal and vertical location uncertainties of 220 m and 400 m, respectively. Holmgren et al.^12^ estimated moment magnitudes ($\:{M}_{w}$) ranging between − 0.4 and 3.8 and a magnitude of completeness ($\:{M}_{c}$) of 1.0. We first discard all earthquakes from 2009 due to absolute timing errors in the waveform data which prevented $\:{M}_{w}$ calculations^12^, resulting in a catalog with 41,151 earthquakes. For the main analysis, we also discard all events below the $\:{M}_{c}$, resulting in a final catalog with 14,882 earthquakes (Fig. 1). Additionally, for the periodicity analysis, we discard 772 shut-in earthquakes identified by Holmgren et al.^12^, who analyzed the same dataset, because they already have a known anthropogenic origin (see “Methods” and Supplementary Fig. S1). Here, we are interested in uncovering new relationships between the operations and reservoir response.

In addition to the seismic data, we use daily injection and production data from 147 wells from the Coso Operating Company, relying on the temperature and cumulative mass injected into the field. Both these data are linearly normalized (see “Methods” and Supplementary Fig. S2 for more details on the temperature normalization).

## Results

### Earthquake periodicity

**Fig. 2:**
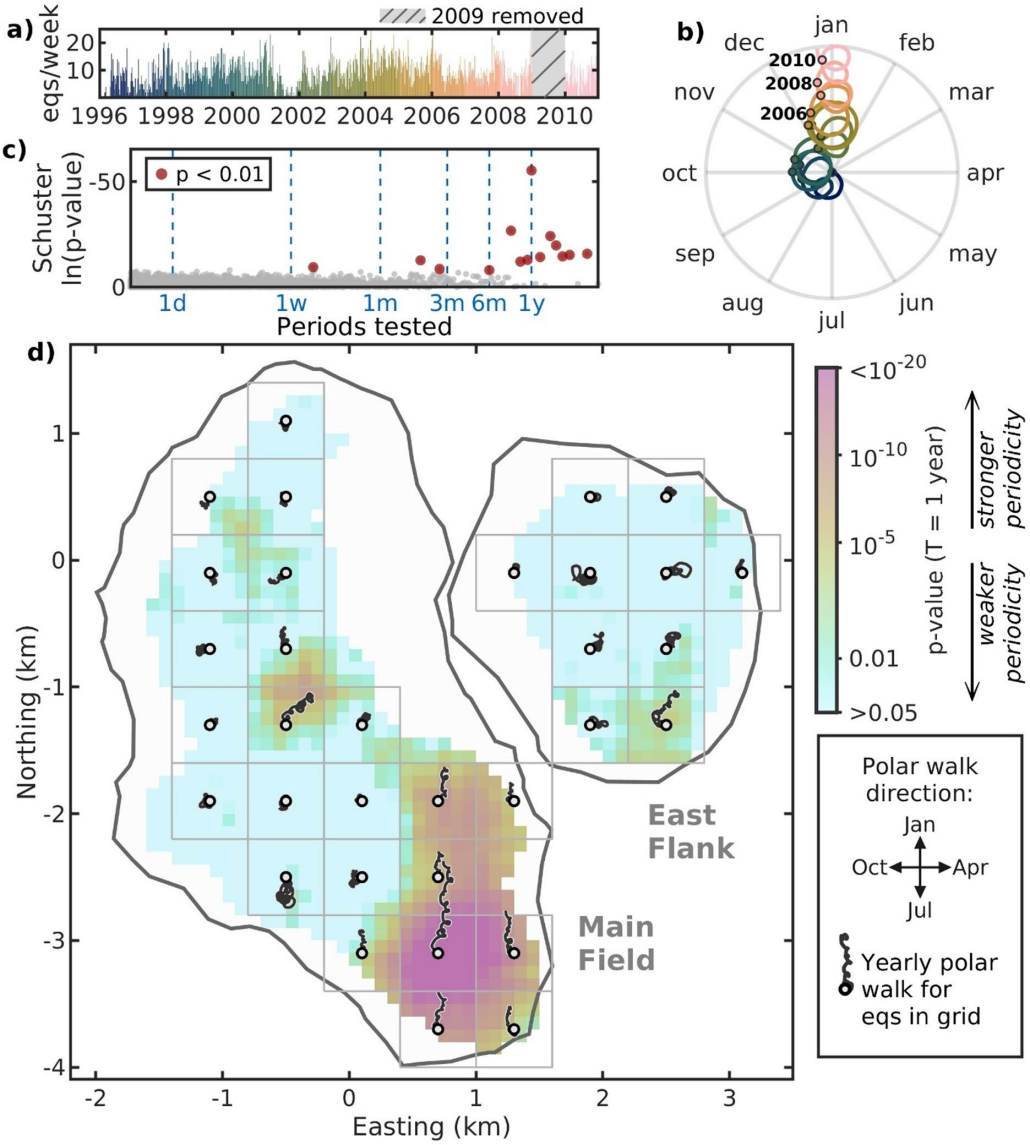
Spatiotemporal seismicity periodicity trends at the Coso Geothermal Field (CGF). (**a**) Weekly earthquake rate after removal of shut-in seismicity from Holmgren et al.^12^ and Nearest-Neighbor Distance (NND) declustering. (**b**) Corresponding Schuster polar walks for period ($\:T$) = 1 year. January 1st of each year is indicated by colored circles outlined in black (years 2006, 2008, and 2010 are labelled). (**c**) Schuster spectrum for the declustered CGF earthquake catalog and resultant $\:p$-value for tested periods between 12 h and 3 years. (**d**) Spatial variations in yearly periodicity of the declustered catalog in (**a**). The heatmap shows the Schuster $\:p$-value for $\:T$ = 1 year of earthquakes within 600-m $\:\times\:$ 600-m bins moved in 100-m increments, requiring a minimum of 75 earthquakes per bin, where the smaller values (darker color) correspond to a stronger detected 1-year periodicity. The grids show the region separated into 600-m $\:\times\:$ 600-m bins with minimum 75 earthquakes and their resultant polar walk for $\:T$ = 1 year. Note the walk lengths are comparable between grids. Map is created using MATLAB^33^ (version R2024b, https://www.mathworks.com) with subfield outlines extracted by clustering the earthquake catalog using DBSCAN^35^ (epsilon neighborhood = 260 m and minimum neighbors = 45).

Periodicity in earthquake catalogs can be identified through the Schuster test and corresponding Schuster spectrum^37,50^, which statistically evaluate whether independent events in a timeseries occur randomly or follow a periodic pattern. Considering the clustered seismicity of the CGF^38^, we first decluster the local earthquake catalog using the Nearest-Neighbor Distance (NND) method^39,40^ to obtain independent events, resulting in a declustered catalog with 6658 earthquakes (Fig. 2a, Supplemental Fig. S3). The Schuster spectrum of the declustered catalog is shown in Fig. 2c, where periods ($\:T$) between 12 h and 3 years are evaluated and $\:T$ with Schuster $\:p$-values below 0.01 (99% confidence level) are highlighted. Because our catalog only spans 15 years, we do not consider $\:T$ above 3 years and instead focus on short-term trends in seismicity rate. Any longer-term relationships between injection trends and seismicity will thus not be detectable through our periodicity analysis. While several $\:T$ resulted in low $\:p$-values, indicating periodicity is detected, only the $\:T$ = 1 year $\:p$-value stands out above the rest. The remaining $\:p$-values below 0.01 (i.e., red circles) are likely due to either the presence of not fully independent events clustering in time (such as swarms or aftershock sequences), implying that some clustering may persist in the catalog despite the declustering process, or sudden bursts of seismicity with duration less than $\:T$^37^. Nonetheless, the clear $\:T$ = 1 year peak indicates a strong annual periodicity in the CGF seismicity. Complementing the Schuster spectrum, we also examine the seismicity’s polar walk for $\:T$ = 1 year (Fig. 2b), which will reveal any temporal preferences within the data for a particular $\:T$. If the walk stays circular near the origin, there is no clear periodicity for the examined $\:T$ = 1 year as this means seismicity rates are consistent throughout the year. However, if the walk successively moves away from the origin in a particular direction, which occurs if seismicity rates are always higher one specific month every year, the direction will indicate the month with heightened seismicity (see “Methods” for more details). The corresponding polar walk for $\:T$ = 1 year (Fig. 2b) reveals a seasonal pattern, where its direction indicates an increased seismicity rate during winter months compared to summer. The polar walk also reveals that the winter periodicity starts in 2003 for the full field, prior to this the walks are more circular in position, suggesting no strong annual trends in seismicity rate. Schuster spectra and polar walks for the full catalog and declustered catalog including shut-in seismicity are shown in Supplemental Figure S4.

To further investigate the spatiotemporal behavior of the CGF’s annual seismic periodicity, we separate the field into smaller grids and assess how the Schuster $\:p$-value varies for $\:T$ = 1 year. The resultant heatmap (Fig. 2d) reveals that the strongest annual periodicity originates in the southern region of the Main Field with a minimum $\:p$-value of $\:1.5\times\:{10}^{-29}$. Furthermore, the periodicity appears to extend 2 km north into the central-eastern Main Field. Examination of the polar walks of earthquakes within the indicated grid cells suggests that this specific area of the field largely drives the winter peak observed in the full-field polar walk (Fig. 2b). Two additional zones exhibiting increased annual periodicity are located in the southern East Flank and central Main Field. However, the seasonal trend in these areas is less distinct, with peak seismicity tending to occur during late winter to early spring rather than mid-winter. Furthermore, upon closer inspection of spatial differences in the Schuster spectrum (Supplemental Fig. S5), these regions’ Schuster $\:p$-value peaks at $\:T$ = 1 year are less distinct in comparison to adjacent periods, thus more likely indicating the presence of bursts of seismicity with duration less than $\:T$ or artifacts of persistent background seismicity rate changes not accounted for by the declustering process^37^. With regards to the shut-in seismicity regions^12^ (see Supplementary Fig. S1), the areas with heightened annual seismic periodicity are located farther east and do not overlap significantly, suggesting that different underlying processes may be driving the seismicity.

### Annual injection trends

**Fig. 3:**
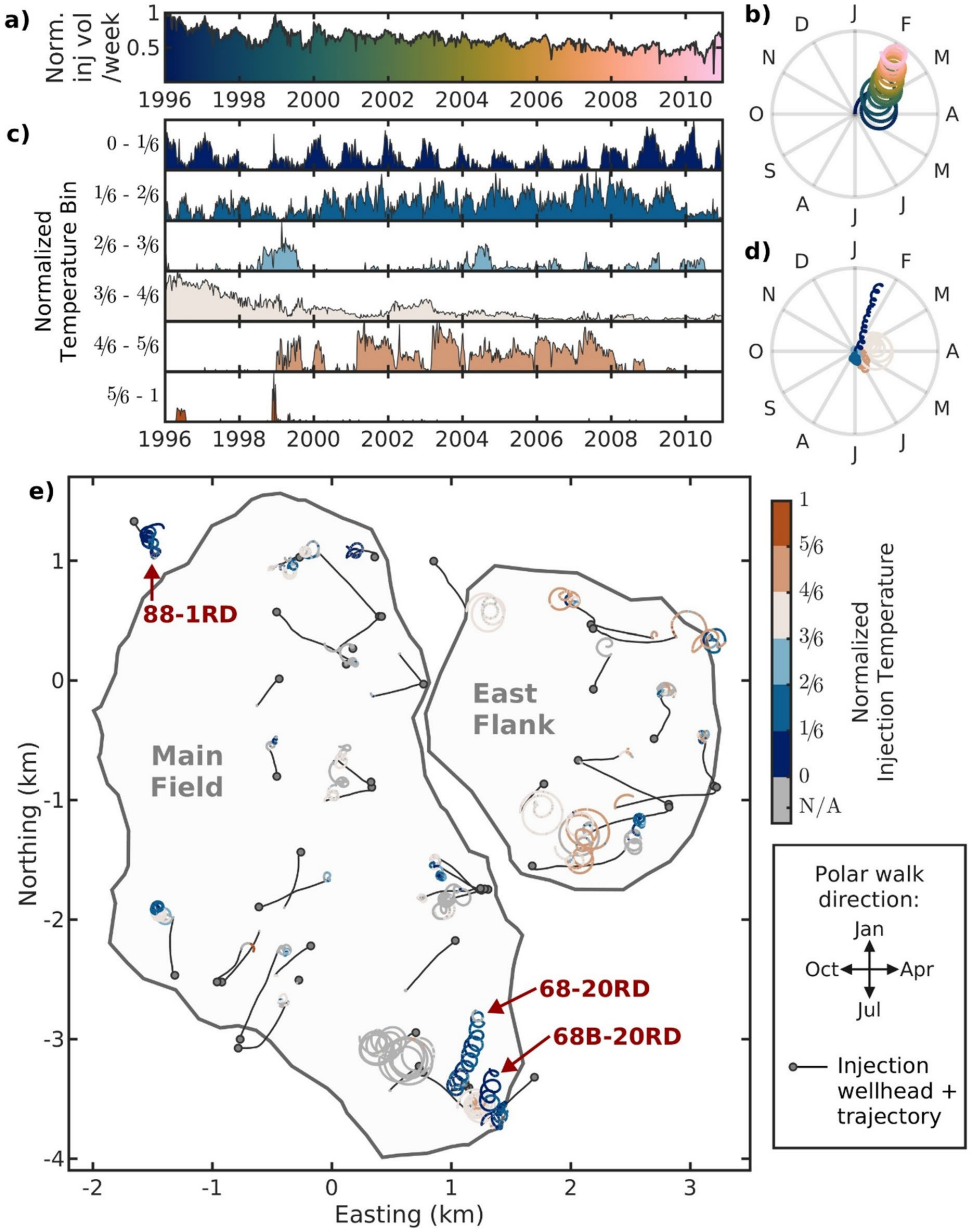
Spatiotemporal injection periodicity trends at the Coso Geothermal Field (CGF). (**a**) Normalized weekly injected volume for the full field. (**b**) Corresponding polar walk for period ($\:T$) = 1 year, colored similarly to (**a**). (**c**) Injected volume in (**a**) separated into six temperature bins (normalized). (**d**) Corresponding polar walks for $\:T$ = 1 year for each temperature bin, colored in the same way as in (**c**). Only the coldest temperature bin (0–1/6) has a strong annual trend. (**e**) Individual wellbore polar walks with $\:T$ = 1 year for all injection wells active during the study period, colored based on the daily temperature recording. N/A (gray) in colorbar is used for days with a recording of injection mass but no injection temperature. The size of the polar walk circles corresponds to the total amount of volume being injected at that well, the larger the circle the more volume is reinjected. Supplementary Figure S2 shows the temperature normalization. Map is created using MATLAB^33^ (version R2024b, https://www.mathworks.com) with subfield outlines extracted by clustering the earthquake catalog using DBSCAN^35^ (epsilon neighborhood = 260 m and minimum neighbors = 45).

We perform a similar polar walk analysis on the injection data to examine any operational links to the annual seismic behavior. The full field’s cumulative injected mass also reveals an annual periodicity, with increased volumes being injected in the winter months (Fig. 3b). When the cumulative mass is separated into six normalized temperature bins (linearly increasing in temperature where 0 = coldest recorded injection and 1 = warmest, see “Methods” and Supplementary Fig. S2 for more details), we find that the lowest 1/6 (i.e., coldest injection bin) represents all the annual periodicity observed in the injection data. This annual periodicity is clear when viewing both the weekly injection volume per temperature bin (Fig. 3c) or their corresponding polar walks for $\:T$ = 1 year (Fig. 3d), where only the coldest temperature bin (dark blue) has a clear walk away from the origin. Larger quantities and colder fluids are injected in wintertime – caused by the lower evaporation rates during winter months^30^. By examining each individual well’s injection patterns through polar walks (Fig. 3e), it becomes apparent that the two injection wells 68-20RD and 68B-20RD in the southern Main Field are the main drivers of the annual winter volumetric periodicity and also reinjected most of the colder condensed steam during the study period. Additionally, injection well 88-1RD north-west of the Main Field also reinjected large quantities of colder fluid, however the injection pattern was not as seasonal. Most of the remaining wells result in circular polar walks, indicating that injection rates are uniform throughout the year or, alternatively, that they do not have clear $\:T$ = 1 year periodicity trends. The same analysis was done on the production data with no clear annual trends observed (Supplemental Fig. S6). Additionally, while the Schuster spectrum cannot be applied to injection time series in the same way it can to an earthquake catalog to identify periodicity, we examine polar walks of the daily injection data using a range of different temporal bins to investigate other periodicities, such as monthly injection patterns (see Supplementary Fig. S7). Annual periodicity is the only one clearly displayed by the injection data.

### Injection vs. Seismicity trends

**Fig. 4:**
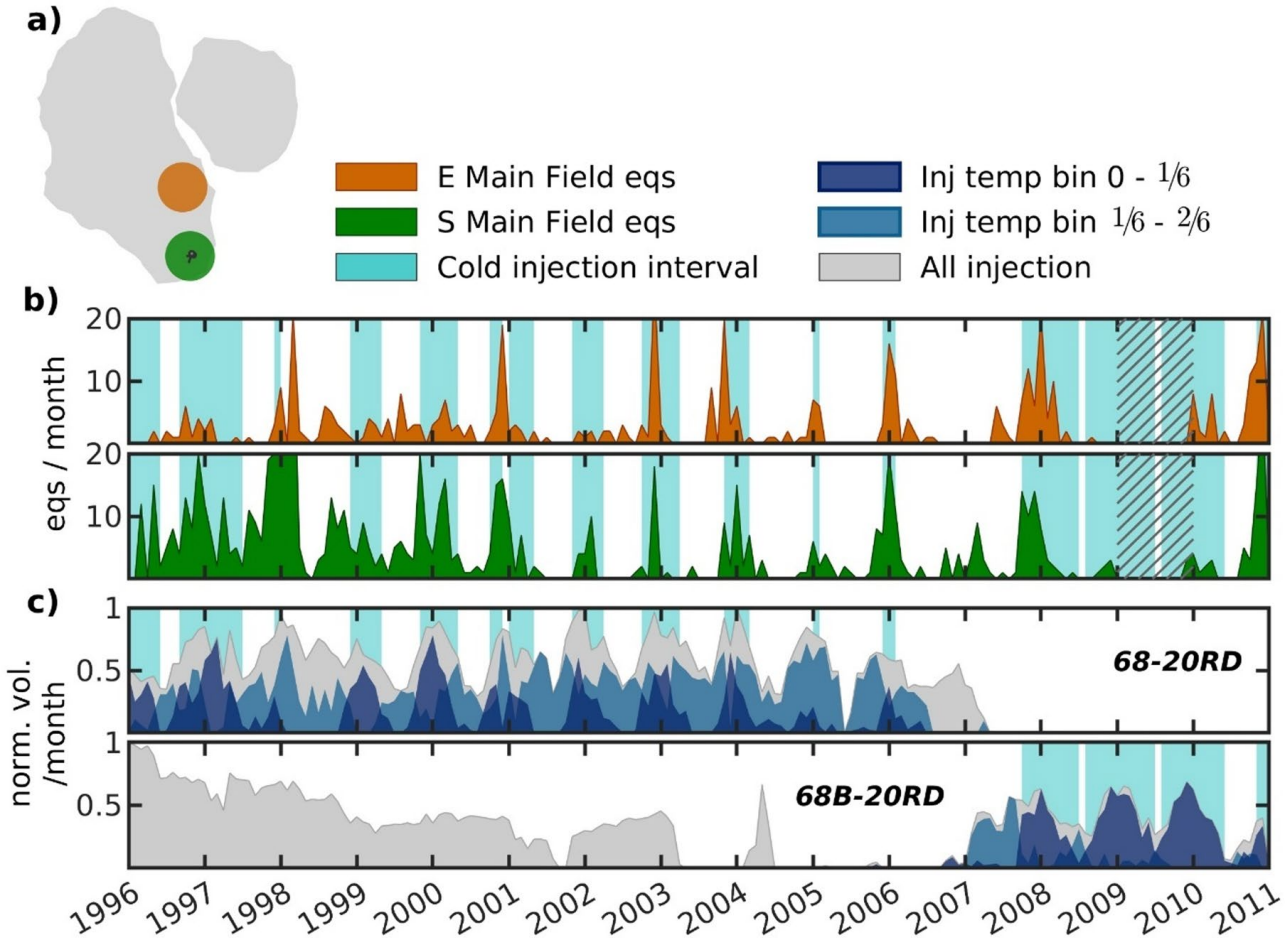
Seismicity ($\:{M}_{w}$ ≥ 1.0, full catalog) and cold injection trends in southern Main Field. (**a**) Map showing two seismicity regions experiencing yearly periodicity, highlighted by 500-m radius areas. Additionally, wellheads and trajectories for 68-20RD and 68B-20RD are shown (black circles and lines, respectively, inside the green area). Note that these wells mostly extend vertically, and so the trajectories appear small. (**b**) Monthly seismicity timeline for the central-eastern and southern Main Field regions (orange and green, respectively) shown in (**a**). Removal of seismicity is indicated by hatched region. Light blue patches indicate cold injection intervals in wellbores 68-20RD or 68B-20RD, where the 0–1/6 temperature bin volume exceeds 10% of their combined maximum monthly injection volume. (**d**) Normalized monthly injection timeline for each of the two wellbores, with injection shown for the two coldest temperature bins (see Supplementary Fig. S2). Map is created using MATLAB^33^ (version R2024b, https://www.mathworks.com) with subfield outlines extracted by clustering the earthquake catalog using DBSCAN^35^ (epsilon neighborhood = 260 m and minimum neighbors = 45).

We investigate the strong annual seismicity trend observed at the Main Field’s southern and central-eastern regions by comparing them to the injection histories of wells 68-20RD and 68B-20RD, both located in the southern Main Field (Fig. 4). Monthly seismicity rates for the full catalog ($\:{M}_{w}$ ≥ 1.0) reveal that both regions experience increased seismicity rates during winter, especially after 1999, with seismicity peaks often coinciding between the regions (Fig. 4b). Furthermore, injection data show that well 68-20RD injected cyclically up until 2007, characterized by peak injection volumes and minimum injection temperatures during winter months. After 2007 when 68-20RD stops injecting, operations at near-by well 68B-20RD continue with a similar injection pattern. It is also noteworthy that 68B-20RD reinjects simultaneously as 68-20RD’s ongoing cyclical injection 1996–2003, although primarily brine at higher temperatures and not the colder condensed steam. To illustrate the injection temperature’s seasonality further, we highlight cold injection intervals in the timelines, defined as months where a minimum of 10% of the monthly injected mass has temperatures recorded within the coldest injection bin (0–1/6), representing time periods with the largest temperature contrast between injection fluid and reservoir.

Seismicity peaks from both regions typically coincide with the initial increase in injection volume and also peak injection volume from the coldest injection bin. The seismicity polar walks of the southern and central-eastern Main Field are oriented towards early January (Fig. 2d), whereas the polar walks for wells 68-20RD and 68B-20RD lag slightly behind (Fig. 3e). This is because the seismicity occurs over shorter time spans, creating spikes in the time series, whereas the injection continues after the seismicity peaks and therefore shifts the polar walk direction (see Supplementary Figure S8 for a zoom-in on weekly injection and seismicity data between 1999 and 2006). An exception to this pattern occurs during the seismicity bursts in early 2006 and in late 2007/early 2008 observed in both regions, which correlates more strongly with a decrease in injection temperature rather than with changes in injection volume, indicating seismicity may be triggered by thermal effects alone during relatively stable injection periods.

We extend the comparison between injection and seismicity trends to the other regions displaying low Schuster $\:p$-values in Fig. 2d, i.e., indicating increased annual seismicity periodicity. When considering the central-eastern Main Field region by itself (Supplementary Fig. S9), seismicity appears to correlate more with the injection patterns from the southern Main Field wells 68-20RD and 68B-20RD (Fig. 4) than with near-by injection wells 67B-17 and 67C-17. Both of the latter primarily reinjected brine (i.e., relatively warmer fluid), with well 67B-17 alternating between brine and condensed steam after 2004. In the southern East Flank (Supplementary Fig. S10), an increased seismicity rate is observed during cold injection intervals at well 86-17, particularly during winter months between 2003 and 2007 when condensed steam is injected. The adjacent well 64A-16, on the other hand, reinjects primarily brine. Finally, the central Main Field shows limited correlation between seismicity and nearby injection activity (Supplementary Fig. S11). This agrees with the absence of a distinct T = 1 year peak in region 5 of the Schuster spectrum (Supplementary Fig. S5), suggesting the presence of bursts of seismicity with duration less than $\:T$ or some persistent change in background seismicity rate is still present in the declustered catalog^37^. All three injection wells near this region primarily reinject brine throughout the study period, with the exception of well 63B-18, which injects mainly condensed steam between mid-1999 and 2003 and experiences an increase in seismicity during the 2001 cold injection.

## Discussion

Here, we observe a clear spatiotemporal correlation between seismicity and injection throughout the CGF using a local seismic network and daily operational data. In particular, we find that the reinjection pattern of the power plant’s output fluid condensed steam can be linked to short-term fluctuations in seismicity rates. This is likely because the field reinjects larger quantities of condensed steam at colder temperatures in the wintertime compared to summertime, creating a periodic pattern in both volume and the temperature contrast to the reservoir. In contrast, geothermal brine is separated from the steam early in the heat extraction process and reinjected at hotter temperatures and at more stable rates throughout the year, thus not inducing temporary seismicity spikes to the same degree. Martínez-Garzón et al.^32^ observed similar features at the CGF temporally, linking increased background and clustered field-wide seismicity rates to heightened injection in winter using a regional earthquake catalog and monthly operational data. At The Geysers geothermal field, Martínez‐Garzón et al.^11^ studied two fluid injection cycles and found peak-fluid injection resulted in a change in the spatiotemporal distribution of the seismicity. Furthermore, correlation between seismicity rate and injection volume and temperature has also been observed at the Hellisheiði geothermal field, Iceland, though only observed during a limited 1-year study period^41^, where an increased intensity in seismicity occurred once injection temperatures dropped below 70 °C^3^. Periodic changes in injection volumes and temperatures hence appear to commonly alter the stress state in conventional geothermal reservoirs, which induces seismicity in distinct patterns as a short-term response.

It is difficult to separate if it is the change in volume or temperature that is the primary triggering mechanism to the observed seismicity trend at the CGF; likely it is a combination of the two effects as increased volume leads to increased pore pressures, whereas the decreased temperature leads to a more rapid cooling near the wells and thermal contraction. Furthermore, the short-term seasonal behavior in seismicity was only observed in the southern Main Field, most likely because the cyclical injection pattern with colder and larger volumes was only carried out at wells 68-20RD and 68B-20RD during our study period 1996–2010. While there were some other wells in the CGF which also injected the colder condensed steam (see Fig. 3 and Supplementary Fig. S9-S11), their injection patterns were more random, making it challenging to link to any nearby seismicity trends. Thus, it is unclear if a similar injection pattern would induce seismicity in a similar pattern elsewhere in the CGF. Likewise, it is unclear if another well injecting, for example, the hotter brine cyclically would create the same seismic response within the reservoir. Instead, we leave that for THM modelers to investigate further and focus here on providing observations from a complex geothermal reservoir.

**Fig. 5:**
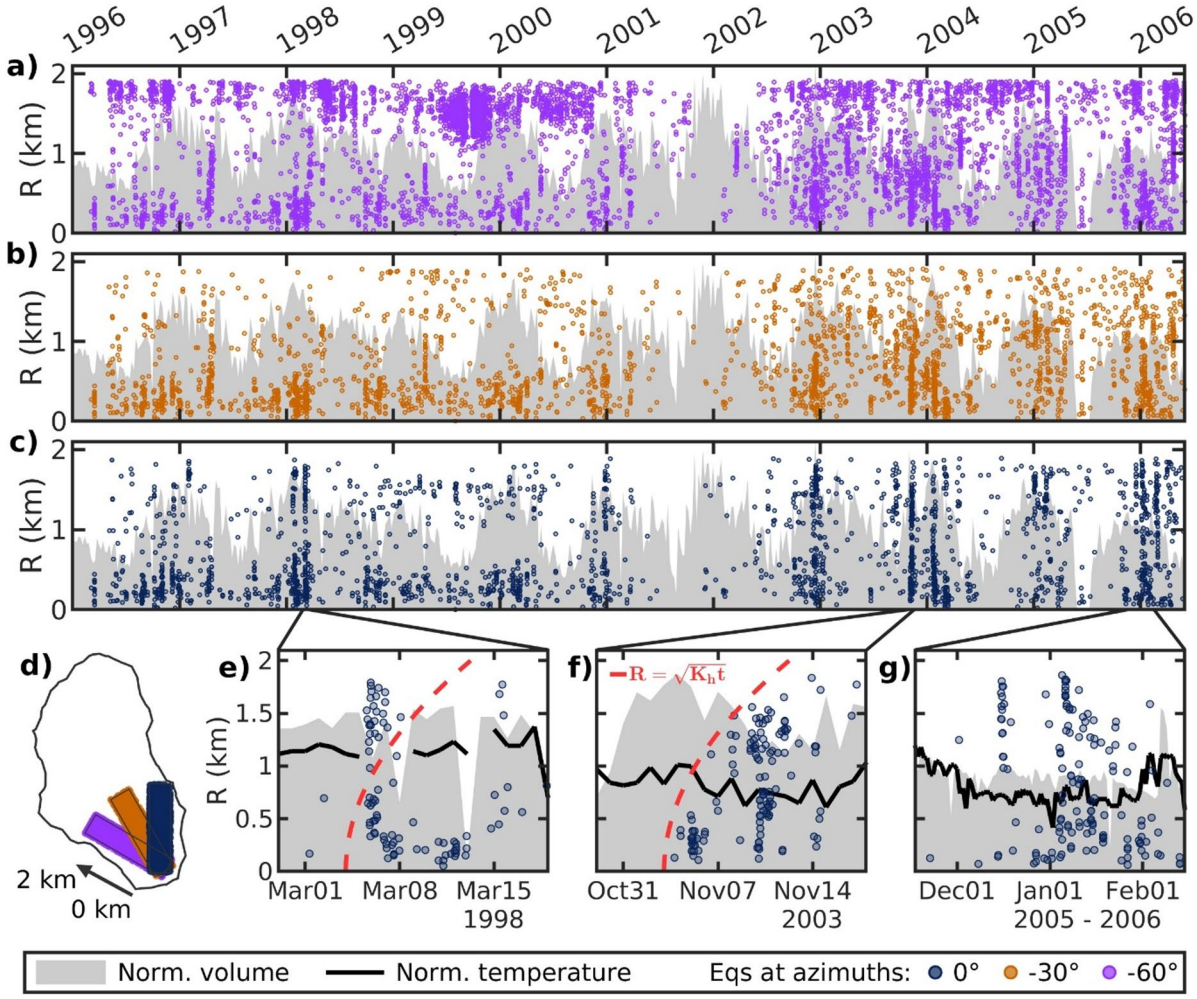
Timeline showing seismicity ($\:{M}_{w}$ ≥ -0.4, circles) with epicentral distance from the 68-20RD wellbore in three directions: (**a**) -60°N, (**b**) -30°N, and (**c**) 0°N. Normalized weekly injection volume from 68-20RD is shown in gray. Note, the catalog is incomplete mid-2001 to mid-2002. (**d**) Map of the Main Field along with the three seismicity bands (2 km $\:\times\:$ 0.5 km) shown in (**a-c**). (**e-g**) Zoom-in on three time periods of the 0°N direction (**c**), showing seismicity occurring along the full 2 km band. Normalized daily injection volume (gray) and, where available, daily injection temperature (black line) from 68-20RD are also shown. The red dashed line in (**e**) and (**f**) shows the hydraulic diffusion distance assuming a hydraulic diffusivity ($\:{K}_{h}$) of 5 m^2^/s. Note that (**e**) and (**f**) both span 20 days, whereas (**g**) spans 90 days. Supplementary Figure S12 shows a similar analysis but for nearby well 68B-20RD. Map is created using MATLAB^33^ (version R2024b, https://www.mathworks.com) with subfield outlines extracted by clustering the earthquake catalog using DBSCAN^35^ (epsilon neighborhood = 260 m and minimum neighbors = 45).

Closer examination of the southern Main Field’s seismic response to increased injection volumes at colder temperatures reveals a preferred directionality in the seismic footprint (Fig. 5). When comparing the injection history of well 68-20RD to seismicity occurring out to 2 km in three different directions, seismic interconnectivity appearing as continuous linear features extending the full 2 km are only obvious towards the north (0°N, Fig. 5c). We further zoom in on three example time periods in the northern direction. Seismic response during March 1998 (Fig. 5e) displays a rapid interconnectivity between the local seismicity and 2 km seismicity, too fast for pore pressure diffusion implying that instantaneous elastic stress transfer can be caused by both pressure and temperature changes, i.e., poro- and/or thermoelastic stress transfer^13,16^. Interestingly, the seismic response in November 2003 displays a seismicity moveout suggesting a hydraulic diffusivity ($\:{K}_{h}$) of 5 m^2^/s (red dashed line, Fig. 5f). For comparison, Martínez-Garzón et al.^11^ estimated a $\:{K}_{h}$ value around 10 m^2^/s for the Geysers Geothermal Field. However, we note the large horizontal location uncertainties in the CGF earthquake catalog (220 m on average) and high magnitude of completeness ($\:{M}_{c}$ = 1.0), likely concealing microseismicity that can shed light on a more robust $\:{K}_{h}$ estimate. Hence, we only include it for discussion. In the third example, we find that the increase in seismicity winter 2005–2006 (Fig. 5g) appears to occur during a relatively stable injection period, thus not linked to any obvious increases in volume. Instead, the seismicity appears to be linked to the decrease in injection temperature, suggesting thermal effects alone can also induce short-term seismicity at distance, likely through cooling close to the well that induces stresses away from the well through elastic stress transfer. Thermoelastic stress influencing the stability of pre-existing faults at far distances beyond the cooling front have been found in THM models^16,42^. A similar trend can be seen for well 68B-20RD (Supplementary Fig. S12).

In contrast to the north-south direction, a similar seismic footprint is not obvious in the other two examined directions in Fig. 5. One of the CGF’s more seismically active regions is found towards the northwest (-60°N, Fig. 5a), as seen from the regular seismicity throughout the study period at distances greater than 1.5 km. Furthermore, tracer tests between injection well 68-20RD and the northwest indicate that there is good connectivity with vapor-phase tracers travelling ~ 1.5 km in just 22 h^43^. Thus, the area likely experiences regular stress release, preventing it from reloading and responding seismically to the changes in stress caused by seasonal injection in the southern Main Field. However, the middle direction (-30°N, Fig. 5b) neither experienced regular seismicity nor displayed a seismic footprint related to the seasonal injection. Unless its stress is released aseismically, this implies that the pore pressure diffusion and poro-thermoelastic stresses are not evenly distributed radially and that there could be structural anisotropy within the CGF. Moreover, as fluid and heat transport in conventional geothermal settings occurs predominantly along faults and fracture, elastic stress transfer from both pore pressure and temperature changes occurs in the fault parallel direction more so than the fault normal direction^3^. Faults surrounding the CGF are mainly oriented north-east or north-west^17,44,45^, however, shear-wave splitting anisotropy analysis from the CGF found north-south fast shear-wave polarizations at a near-by seismic station^46^, which could indicate there are north-south faults within the area and explain why we see the north-south seismic footprint. Additionally, Martínez-Garzón et al.^11^ found that seismicity during peak injection reached further distances in direction parallel to the maximum horizontal stress at the Geysers. The maximum horizontal stress at the CGF is roughly north-south ± 20°, which also agrees with our observed north-south seismic footprint, although in-situ borehole measurements indicating large heterogeneity within the field and at times local stress field rotations by 90° were observed^45,47^.

The Schuster test assumes the seismicity examined is independent in time, thus earthquake catalogs require declustering before analysis. Furthermore, when assessing a long-term producing geothermal field such as the CGF, there is also the consideration of longer-term changes in operations or reservoir characteristics which could affect seismicity rates. While the $\:T$ = 1 year seismic periodicity can be clearly linked to the injection patterns at the southern Main Field, the Schuster spectrum also detected several low Schuster $\:p$-values for $\:T$ up to three years (Fig. 2c), which could indicate periodicity associated with longer-term changes in operation. However, because these $\:T$ with low $\:p$-values are not as clear as $\:T$ = 1 year, they are likely caused by bursts of seismicity with duration less than $\:T$^37^. An earthquake catalog spanning more than 15 years would provide more data at these longer periods and could potentially shed better insight on long-term periodicity trends.

Our case study analyzing 15 years of local seismic and daily operation data reveals that short-term seismic response at the CGF can be linked to seasonal variations in the reinjection of colder condensed steam rather than the hotter brine, which is injected at more stable rates year round. Furthermore, the volumetric increase and temperature decrease of injection fluids in wintertime produces poro- and/or thermoelastic stressing that concentrates north-south, triggering earthquakes near-instantaneously up to 2 km away from the injection well too rapidly for pore pressure diffusion. In contrast, regions towards the north-west do not respond seismically to the seasonal injection pattern, indicating a strong directional preference in the seismic footprint and possible structural or permeability anisotropy within the CGF. Finally, the bursts of seismicity with a 2 km footprint correlate with changes in volume and temperature combined, but also with temperature decrease alone during relatively stable injection rates. This suggests that thermal effects can play an important role in triggering short-term seismic responses at a distance from the injection well, not just pressure effects. These findings further suggest that fluid and heat transfer in conventional, naturally fractured and faulted geothermal reservoirs occur on various scales in space and time; major fluid and heat pathways with fast transport control certain parts of the field, while distributed, slower transport of fluids and heat occurs in other regions. This indicates separation of physical processes and their timescales in these natural settings and hints at the special geologic settings required to sustain conventional geothermal reservoirs. Detailed analysis of the interplay between injection and induced seismicity sheds important insight into the hydro-thermal characteristics of geothermal fields. For example, periodic cold fluid injections may aid with improving or maintaining permeability along fractures, providing guidance in the design of injection strategies for long-term heat production at the CGF and other fields.

## Methods

### Temperature data normalization

We normalize the production and injection temperatures from the Coso Operating Company because the data are proprietary. First, we initially normalize all daily temperature recordings by dividing them by temperature $\:{T}_{norm}$ so that 0.0 = 0 °C and 1.0 = $\:{T}_{norm}$, see Supplementary Figure S2a. We chose $\:{T}_{norm}$ so that it is larger than the production’s daily temperature recordings (only excluding a few extreme outliers), ensuring the span 0 °C to $\:{T}_{norm}$ covers both the injection and production temperatures. As can be seen, the injection temperature recordings follow a bimodal distribution, where the lower peak corresponds to the reinjected colder condensed steam and the higher peak is the reinjected hotter brine (see Fig. 1c). Next, we separate the normalized temperature data into eight equal bins, finding that the injection temperature recordings span the first six bins (Supplementary Fig. S2b) and the production temperature recordings span the last five bins (Supplementary Fig. S2c). Thus, we use these six and five bins to represent our normalized injection and production data, respectively. For the injection temperatures, 0.0 to 1.0 span the initial 0.0 to $\:{T}_{norm}\times\:$ 0.75. For the production temperatures, 0.0 to 1.0 span $\:{T}_{norm}\times\:$ 0.375 to $\:{T}_{norm}$. The temperature bins are used to separate the injection and production volume data based on temperature to better highlight seasonal trends in Figs. 3 and 4 and Supplementary Figures S6 and S9-S11.

### Declustering earthquake catalogue

In order to examine spatiotemporal trends in the CGF, we decluster the earthquake catalog to isolate the independent seismicity. Before declustering, we remove all earthquakes below the magnitude of completeness ($\:{M}_{c}=1.0$^12^. Additionally, we remove the shut-in earthquakes reported by Holmgren et al.^12^ as these have known anthropogenic sources. Next, we decluster the CGF earthquake catalog using the Nearest-Neighbor Distance (NND) technique^39,40^, which does not assume a specific form of earthquake clustering and is suitable for geothermal fields dominated by seismic swarms^32^. NND relies on the space-time-magnitude distance $\:{\eta\:}_{ij}$ between earthquakes $\:i$ and $\:j$, which is defined as:

1 $$\:{\eta\:}_{ij}=\left\{\begin{array}{c}{t}_{ij}\:{r}_{ij}^{d}\:{10}^{-10b{m}_{i}},\:\:{t}_{ij}>0\\\:\infty\:,\:\:\:\:\:\:\:\:\:\:\:\:\:\:\:\:\:\:\:\:\:\:\:\:\:\:\:\:\:\:\:\:\:\:{t}_{ij}\le\:0\end{array}\right.$$

where $\:{t}_{ij}={t}_{j}-{t}_{i}$ is the interevent time in years, $\:{r}_{ij}$ is the interevent distance (epicentral) in km, $\:d$ is the fractal dimension that expresses the spatial distribution of earthquakes, $\:b$ is the $\:b$-value from the earthquake magnitude-frequency distribution, and $\:{m}_{i}$ is the magnitude of event $\:i$. We set $\:d$ = 1.8 following Schoenball et al.^38^ who performed a similar NND analysis using a CGF regional earthquake catalog. The $\:b$-value is estimated using the $\:{b}^{+}$ approach^48^ (Supplementary Fig. S3a). Using the full CGF catalog and a magnitude difference cut-off ($\:{\Delta\:}{M}_{w}$) of 0.40, we find that $\:{b}^{+}$ = 1.48 ± 0.02, which is consistent with higher $\:b$-values commonly found for fluid-induced seismicity^38^. We note that while other studies have found CGF $\:b$-values of 1.34 ± 0.24^44^, 1.14 ± 0.11^38^, 1.2 to 1.3^31^, and 0.94 ± 0.02^32^, they relied on coda-length magnitudes or the local magnitudes reported in the Southern California Earthquake Catalogue, whereas we rely on the $\:{M}_{w}$ estimated by Holmgren et al.^12^. Different magnitude scales have been shown to result in different $\:b$-values^49^. Furthermore, similar to Schoenball et al.^38^, we find that varying the $\:d$ and $\:b$-value does not affect the $\:{\eta\:}_{ij}$-distribution significantly. The $\:{\eta\:}_{ij}$ distribution can also be separated into its rescaled space and time components $\:{\eta\:}_{ij}={T}_{ij}{R}_{ij}$, where:

2 $$\:{T}_{ij}={t}_{ij}{10}^{-b{m}_{i}/2}$$

3 $$\:{R}_{ij}={r}_{ij}^{d}{10}^{-b{m}_{i}/2}$$

Once $\:{\eta\:}_{ij}$ have been estimated for all event-pairs, the nearest neighbor event $\:i$ for each earthquake $\:j$ is found through $\:{\eta\:}_{j}=\underset{i}{\mathrm{min}}{n}_{ij}$. Then, the frequency histograms for $\:\eta\:$ and ($\:T$, $\:R$) (Supplementary Fig. S3c and S3d) can be used to identify the two modes representing the background and clustered seismicity within the catalog. Similar to Schoenball et al.^38^, we find a strong presence of background events reoccurring within close proximity of each other (small $\:R$ and large $\:T$), likely representing fractures consistently reactivated over time after being reloaded by the geothermal field’s long-term production activity. We obtain the separation between the modes ($\:{\eta\:}_{0}$) by fitting the $\:{\mathrm{log}}_{10}\eta\:$ distribution to a Gaussian mixture model and finding the intersect between the two resultant Gaussian distributions (Supplementary Fig. S3c), resulting in $\:{\mathrm{log}}_{10}{\eta\:}_{0}$ = -6.72 for the local CGF catalog. Any earthquakes with weak links (i.e., $\:{\eta\:}_{j}$ > $\:{\eta\:}_{0}$) are labelled *single* events. The remaining events are referred to as clustered events, which are further separated into individual clusters through graph-based clustering and connected components using $\:{\eta\:}_{0}$ as the cutoff. The largest event in each cluster is labelled *mainshock* and the rest *offspring*. The final declustered CGF catalogue of independent events consists of all the *single* events and *mainshock* events, which results in a CGF catalog with 6658 earthquakes.

### Schuster test and Polar walks

We investigate periodicity in the CGF through Schuster tests^50^, which have been previously applied to earthquake catalogs to identify seismicity modulation by, for example, solid-earth tides^26^ and anthropogenic activities^51^. Assuming an earthquake catalog consists of independent event times $\:{t}_{k}$, the phase $\:{\theta\:}_{k}$ of event $\:k$ with respect to a period $\:T$ is:

4 $$\:{\theta\:}_{k}=2\pi\:\frac{{t}_{k}}{T}\:,$$

where the unit of $\:T$ and $\:{t}_{k}$ are the same. We can then convert the catalogue times into a polar walk (2D random walk) made of successive unit-length steps with each event’s direction given by $\:{\theta\:}_{k}$ (e.g., Fig. 2b). Simply put, if an earthquake occurred on January 1st, it would take a step with length = 1 towards $\:{\theta\:}_{k}$ = 0° in a polar plot, where 0° to 360° represents January 1st to December 31st. We can then calculate $\:D$, which is the distance between the first and final walk points and can be estimated through

5 $$\:D=\sqrt{{\left({\sum\:}_{k=1}^{N}\mathrm{cos}{\theta\:}_{k}\right)}^{2}+{\left({\sum\:}_{k=1}^{N}\mathrm{sin}{\theta\:}_{k}\right)}^{2}}\:,$$

where $\:N$ is the number of events. This allows us to find the Schuster $\:p$-value, which expresses the probability that the event times come from a uniform seismicity rate (i.e., the null hypothesis):

6 $$\:p=\mathrm{exp}\left(-\frac{{D}^{2}}{N}\right)\:$$

Generally, if $\:p$ ≤ 0.05, the null hypothesis is rejected and the earthquake catalog exhibits periodicity for the period $\:T$. Ader and Avouac^37^ pointed out that the Schuster test alone may not be enough to indicate periodicity because a low $\:p$-value can be obtained by both a seismicity rate with periodicity $\:T$ and a sudden burst of seismicity with duration less than $\:T$. For example, if $\:T$ = 1 year and a burst 100 seismic events occurs over one week during one of the years, resulting in 100 extra unit length steps in that $\:{\theta\:}_{k}$ direction, then it will lead to a larger $\:D$ and a smaller $\:p$-value (Eq. 6). To resolve this, they extended the Schuster test and proposed the Schuster spectrum. By testing a range of $\:T$ instead of just one, the Schuster spectrum can identify if a catalog’s periodicity is unique. We calculate the Schuster spectrum using the NND declustered CGF catalog, identifying $\:T$ = 1 year as the most prominent seismicity rate periodicity (Fig. 2c). The Schuster spectrum and resultant polar walks for the full catalog and NND-declustered including shut-in seismicity is shown in Supplementary Fig. S4. Here, the polar walk for the full catalog (Supplementary Fig. S4a) shows an example burst of seismicity in early 2010 which is shorter than $\:T$ = 1 year and creates an almost straight line in the walk, increasing the final $\:D$ estimate (distance between first and last polar walk) and decreasing the Schuster $\:p$-value.

In this study, we discard all earthquakes from 2009 due to absolute timing errors which led to no moment magnitudes calculated by Holmgren et al.^12^ and earthquakes that were labelled shut-in events by Holmgren et al.^12^ as these already had a known anthropogenic origin (maintenance shut-ins in springtime). For this analysis, we are interested in periodicity of earthquakes with unknown origin to detect new relationships between operations and reservoir response. Thus, if we were to keep the shut-in events, the polar walks would be biased and shifted towards months when the shut-in events occur. To test if removing a year or specific events each year affects the Schuster test or polar walks, we create a synthetic catalog with annual periodicity between 1996 and 2010 (Supplementary Figure S13). We use Ogata’s modified thinning algorithm^52^ to first simulate a homogeneous Poisson process to obtain a synthetic catalog of temporally independent earthquakes, followed by thinning the points in time to ensure the earthquake rate varies annually. As can be seen in Supplementary Figure S13b, removing one year does not significantly affect the periodicity results nor introduce any new periodicities. With regards to the shut-in seismicity, the CGF has four power plants that control a portion of the field’s wellbores each and generally one power plant is shut in at a time during the annual springtime maintenance. Holmgren et al.^12^ labelled any event occurring within the spatial extent of the wellbores whose power plant was shut in as a shut-in earthquake. To simplify this for the synthetic test, we remove seismicity occurring on any day labelled shut in by any of the power plants from the simulated catalog, resulting in 34 days between 1996 and 2010. As can be seen in Supplementary Figure S13c, this results in a very minor anticlockwise shift in the polar walk. Comparison between retaining and removing shut-in earthquakes from the CGF declustered catalog (Supplementary Fig. S4b and S4c, respectively) reveals a much larger anticlockwise shift when shut-in seismicity is excluded. This shift highlights the bursts of seismicity caused by the shut-ins and the importance in removing them before analyzing the polar walks to be able to focus on unknown seismicity trends.

For the operational data, we plot the polar walks of the cumulative mass of both the full field and individual wells. Instead of assuming that each 2D random walk has a unit step length, we let the step length be equal to the day’s cumulative mass.

## Supplementary Information

Below is the link to the electronic supplementary material.

Supplementary Material 1

## Data Availability

The data that support the findings of this study are available from the U.S. Navy Geothermal Program Office and the Coso Operating Company, but restrictions apply to the availability of these data, which were used under license for the current study and are not publicly available. Data are, however, available from the authors upon reasonable request and with the permission of the U.S. Navy Geothermal Program Office and the Coso Operating Company. California outline in Figure 1a is extracted using the borders function from Greene et al.^34^.

## References

[1] De Simone, S., Carrera, J. & Vilarrasa, V. Superposition approach to understand triggering mechanisms of post-injection induced seismicity. *Geothermics* **70**, 85–97 (2017).

[2] Jeanne, P. et al. Reservoir structure and properties from Geomechanical modeling and microseismicity analyses associated with an enhanced geothermal system at the Geysers, California. *Geothermics* **51**, 460–469 (2014).

[3] Cao, W. et al. Induced seismicity associated with geothermal fluids re-injection: poroelastic stressing, thermoelastic stressing, or transient cooling-induced permeability enhancement? *Geothermics* **102**, 102404 (2022).

[4] White, M. C., Nakata, N., Tribaldos, V. R., Nayak, A. & Dobson, P. F. Seismotectonic evolution and geothermal energy production in the Salton Sea Geothermal Field. Proc. 48th Workshop on Geothermal Reservoir Engineering (Stanford Univ., Stanford, California, 6–8 February 2023). SGP-TR-224 (2023).

[5] Grant, M. A., Clearwater, J., Quinão, J., Bixley, P. F. & Le Brun, M. Thermal stimulation of geothermal wells: a review of field data. Proc. 38th Workshop on Geothermal Reservoir Engineering (Stanford Univ., Stanford, California, 11–13 February 2013) SGP-TR-198 (2013).

[6] Rawal, C. & Ghassemi, A. A reactive thermo-poroelastic analysis of water injection into an enhanced geothermal reservoir. *Geothermics* **50**, 10–23 (2014).

[7] Izadi, G. & Elsworth, D. The influence of thermal-hydraulic-mechanical-and chemical effects on the evolution of permeability, seismicity and heat production in geothermal reservoirs. *Geothermics* **53**, 385–395 (2015).

[8] Moein, M. J. et al. The physical mechanisms of induced earthquakes. *Nat. Rev. Earth Environ.* **4**, 847–863 (2023).

[9] Jiang, G. et al. Relatively stable pressure effects and time-increasing thermal contraction control Heber geothermal field deformation. *Nat. Commun.* **15**, 5159 (2024). 38886394 10.1038/s41467-024-49363-1 PMC11183258

[10] Koirala, R. et al. Induced seismicity and surface deformation associated with long-term and abrupt geothermal operations in blue Mountain, Nevada. *Earth Planet. Sci. Lett.* **643**, 118883 (2024).

[11] Martínez-Garzón, P. et al. Spatiotemporal changes, faulting regimes, and source parameters of induced seismicity: A case study from the geysers geothermal field. *J. Geophys. Res. Solid Earth*. **119**, 8378–8396 (2014).

[12] Holmgren, J. M., Kaven, J. O. & Oye, V. Long-Term trends in microseismicity during operational Shut-Ins at the Coso geothermal Field, California. *Seismic Record*. **5**, 73–82 (2025).

[13] Rutqvist, J. et al. The Northwest geysers EGS demonstration project, california: pre-stimulation modeling and interpretation of the stimulation. *Math. Geosci.* **47**, 3–29 (2015).

[14] Goebel, T. H. & Brodsky, E. E. The Spatial footprint of injection wells in a global compilation of induced earthquake sequences. *Science* **361**, 899–904 (2018). 30166486 10.1126/science.aat5449

[15] Boyet, A., De Simone, S., Ge, S. & Vilarrasa, V. Poroelastic stress relaxation, slip stress transfer and friction weakening controlled post-injection seismicity at the Basel enhanced geothermal system. *Commun. Earth Environ.* **4**, 104 (2023).

[16] Kivi, I. R., Pujades, E., Rutqvist, J. & Vilarrasa, V. Cooling-induced reactivation of distant faults during long-term geothermal energy production in hot sedimentary aquifers. *Sci. Rep.* **12**, 2065 (2022). 35136121 10.1038/s41598-022-06067-0 PMC8826403

[17] Bhattacharyya, J. & Lees, J. M. Seismicity and seismic stress in the Coso Range, Coso geothermal field, and Indian Wells Valley region, southeast-central California. in *Geologic Evolution of the Mojave Desert and Southwestern Basin and Range* (eds. A. F. Glazner, J. D. Walker, & J. M. Bartley) (2002). 10.1130/0-8137-1195-9.243

[18] Hauksson, E. & Unruh, J. Regional tectonics of the Coso geothermal area along the intracontinental plate boundary in central Eastern california: Three-dimensional Vp and Vp/Vs models, spatial‐temporal seismicity patterns, and seismogenic deformation. *J. Geophys. Res. Solid Earth*. **112**, B06309 (2007).

[19] Bacon, C. R., Duffield, W. A. & Nakamura, K. Distribution of quaternary rhyolite domes of the Coso Range, california: implications for extent of the geothermal anomaly. *J. Geophys. Res. Solid Earth*. **85**, 2425–2433 (1980).

[20] Wamalwa, A. M., Mickus, K. L., Serpa, L. F. & Doser D. I. A joint geophysical analysis of the Coso geothermal Field, south-eastern California. *Phys. Earth Planet. Inter*. **214**, 25–34 (2013).

[21] Zhang, Q. & Lin, G. Three-dimensional Vp and Vp/Vs models in the Coso geothermal area, california: seismic characterization of the magmatic system. *J. Geophys. Res. Solid Earth*. **119**, 4907–4922 (2014).

[22] Im, K., Avouac, J. P., Heimisson, E. R. & Elsworth, D. Ridgecrest aftershocks at Coso suppressed by thermal destressing. *Nature* **59**, 70–74 (2021). 10.1038/s41586-021-03601-4 34194023

[23] Tung, S. et al. Seismicity zoning at Coso geothermal field and stress changes from fluid production and migration. *Earth Planet. Sci. Lett.* **646**, 119000 (2024).

[24] Cardiff, M. et al. Geothermal production and reduced seismicity: correlation and proposed mechanism. *Earth Planet. Sci. Lett.* **482**, 470–477 (2018).

[25] Guo, H. et al. Microseismicity modulation due to changes in geothermal production at San Emidio, Nevada, USA. *Geophys. Res. Lett.* **52**, eGL112063 (2025).

[26] Wang, W. et al. Tidal modulation of seismicity at the Coso geothermal field. *Earth Planet. Sci. Lett.* **579**, 117335 (2022).

[27] Hill, D. P. et al. Seismicity remotely triggered by the magnitude 7.3 Landers, California, earthquake. *Science* **260**, 1617–1623 (1993). 17810202 10.1126/science.260.5114.1617

[28] Aiken, C. & Peng, Z. Dynamic triggering of microearthquakes in three geothermal/volcanic regions of California. *J. Geophys. Res. Solid Earth*. **119**, 6992–7009 (2014).

[29] Harvey, W. & Wallace, K. Flash steam geothermal energy conversion systems: single-, double-, and triple-flash and combined-cycle plants. In Geothermal Power Generation 249–290Woodhead Publishing, (2006).

[30] Buck, C. The Role of Ammonia at the Coso Geothermal Field. 50th Workshop on Geothermal Reservoir Engineering (Stanford Univ., Stanford, California, 10–12 February 2025). SGP-TR-229 (2025).

[31] Trugman, D. T., Shearer, P. M., Borsa, A. A. & Fialko, Y. A comparison of long-term changes in seismicity at the Geysers, Salton Sea, and Coso geothermal fields. *J. Geophys. Res. Solid Earth*. **121**, 225–247 (2016).

[32] Martínez-Garzón, P., Zaliapin, I., Ben‐Zion, Y., Kwiatek, G. & Bohnhoff, M. Comparative study of earthquake clustering in relation to hydraulic activities at geothermal fields in California. *J. Geophys. Res. Solid Earth*. **123**, 4041–4062 (2018).

[33] The MathWorks Inc. MATLAB version 24.2.0.2863752 (R2024b) Update 5, Natick, Massachusetts: The MathWorks Inc. (2024). https://www.mathworks.com

[34] Greene, C. A. et al. The Climate Data Toolbox for MATLAB. Geophysics, Geosystems doi:10.1029/2019GC008392 (2019).

[35] Ester, M., Kriegel, H. P., Sander, J. & Xiaowei, X. A density-based algorithm for discovering clusters in large spatial databases with noise. In Proceedings of the Second International Conference on Knowledge Discovery in Databases and Data Mining, 226–231. Portland, OR: AAAI Press (1996).

[36] Kaven, J. O., Hickman, S. H. & Davatzes, N. C. Microseismicity within the Coso Geothermal Field, California, from 1996 to 2012. Proc. 38th Workshop on Geothermal Reservoir Engineering (Stanford Univ., Stanford, California, 11–13 February 2013). SGP-TR-198 (2013).

[37] Ader, T. J. & Avouac, J. P. Detecting periodicities and declustering in earthquake catalogs using the Schuster spectrum, application to Himalayan seismicity. *Earth Planet. Sci. Lett.* **377**, 97–105 (2013).

[38] Schoenball, M., Davatzes, N. C. & Glen, J. M. Differentiating induced and natural seismicity using space-time‐magnitude statistics applied to the Coso geothermal field. *Geophys. Res. Lett.* **42**, 6221–6228 (2015).

[39] Zaliapin, I. & Ben-Zion, Y. Earthquake clusters in Southern California I: identification and stability. *J. Geophys. Res. Solid Earth*. **118**, 2847–2864 (2013).

[40] Zaliapin, I. & Ben-Zion, Y. Earthquake declustering using the nearest‐neighbor approach in space‐time‐magnitude domain. *J. Geophys. Res. Solid Earth* 125, e2018JB017120 (2020).

[41] Gunnarsson, G. Temperature dependent injectivity and induced Seismicity–Managing reinjection in the Hellisheiði Field, SW-Iceland. *Geotherm. Resour. Counc. Trans.* **37**, 1020–1025 (2013).

[42] Jeanne, P. et al. The impacts of mechanical stress transfers caused by hydromechanical and thermal processes on fault stability during hydraulic stimulation in a deep geothermal reservoir. *Int. J. Rock. Mech. Min. Sci.* **72**, 149–163 (2014).

[43] Mella, M., Rose, P., McCulloch, J. & Buck, C. A tracer test using ethanol as a two-phase tracer and 2-naphthalene sulfonate as a liquid-phase tracer at the Coso geothermal field. *Geotherm. Resour. Counc. Trans.* **30**, 1–3 (2006).

[44] Walter, A. W. & Weaver, C. S. Seismicity of the Coso range, California. *J. Geophys. Res. Solid Earth*. **85**, 2441–2458 (1980).

[45] Davatzes, N. C. & Hickman, S. Stress and faulting in the Coso Geothermal Field: Update and recent results from the East Flank and Coso Wash. Proc. 31st Workshop on Geothermal Reservoir Engineering (Stanford Univ., Stanford, California, 30 January – 1 February 13–19 (2006). (2006).

[46] Vlahovic, G., Elkibbi, M. & Rial, J. A. Shear-wave splitting and reservoir crack characterization: the Coso geothermal field. *J. Volcanol Geotherm. Res.* **120** (1–2), 123–140 (2003).

[47] Schoenball, M. & Davatzes, N. C. Quantifying the heterogeneity of the tectonic stress field using borehole data. *J. Geophys. Res. Solid Earth*. **122**, 6737–6756 (2017).

[48] van der Elst, N. J. B-positive: A robust estimator of aftershock magnitude distribution in transiently incomplete catalogs. *J. Geophys. Res. Solid Earth* 126, e2020JB021027 (2021).

[49] Baltay, A. & Abercrombie, R. E. Seismic moment and local magnitude scales in Ridgecrest, California, from the SCEC/USGS community stress drop validation study. *Bull. Seismol. Soc. Am.* **115**, 1279–1293 (2025).

[50] Schuster, A. On the investigation of hidden periodicities with application to a supposed 26 day period of meteorological phenomena. *Terr. Magn.* **3**, 13–41 (1898).

[51] Acosta, M. et al. Earthquake nucleation characteristics revealed by seismicity response to seasonal stress variations induced by gas production at Groningen. *Geophys. Res. Lett.* **50**, e2023GL105455 (2023).

[52] Ogata, Y. On lewis’ simulation method for point processes. *IEEE Trans. Inf. Theory*. **27**, 23–31 (1981).

