# Supplementary material for: Rapid hydrothermal triggering of induced seismicity at the Coso geothermal field
Source: Sci Rep. 2026 Feb 3;16:7057. doi: 10.1038/s41598-026-38146-x (PMC12920652; doi:10.1038/s41598-026-38146-x)
Supplement: Supplementary file 1 — Supplementary Material 1 [file 41598_2026_38146_MOESM1_ESM.pdf]

Supplementary information to Holmgren et al. (2026), *Rapid hydrothermal triggering of induced seismicity at the Coso Geothermal Field*

Contents of this file

Figure S1: Magnitude timeline and map of shut-in seismicity from Holmgren et al. (2025), which is removed from this analysis.

Figure S2: Temperature normalization scheme for the operational temperature data, showing the bins used for injection and production.

Figure S3: Nearest-neighbor distance (NND) declustering overview. Figure shows the b^+ value and NND space-time-magnitude distance histograms.

Figure S4: Schuster analysis and polar walks for full catalog, NND declustered catalog including shut-in (SI) events, and SI events removed prior to NND declustering.

Figure S5: Spatial variation in Schuster spectrum for the CGF. The CGF is separated into 6 bins of similar size, and their resultant Schuster spectra are shown.

Figure S6: Spatiotemporal production periodicity trends at the CGF (similar to Figure 3, but for production data). Figure shows the production data separated into temperature bins, and also each well's polar walk in spatial view.

Figure S7: Examination of daily injection volume polar walks for six different periods: 1 month, 2 months, 3 months, 6 months, 1 year, and 2 years.

Figure S8. Zoom-in of injection temperature and volume for well 68-20RD between 1999 and 2006 (peak periodicity years) along with near-by seismicity.

Figures S9-S11: Seismicity and cold injection trends in central-eastern Main Field (Fig. S9), southern East Flank (Fig. S10), and central Main Field (Fig. S11), similar to Figure 4.

Figure S12: Timeline showing seismicity with epicentral distance from the 68B-20RD wellbore in three directions: -60°N, -30°N, and 0°N.

Figure S13: Synthetic testing how the Schuster spectrum and periodicity is affected by removing one full year or a few days each spring from a synthetic earthquake catalog with a strong annual periodicity between 1996 and 2010.

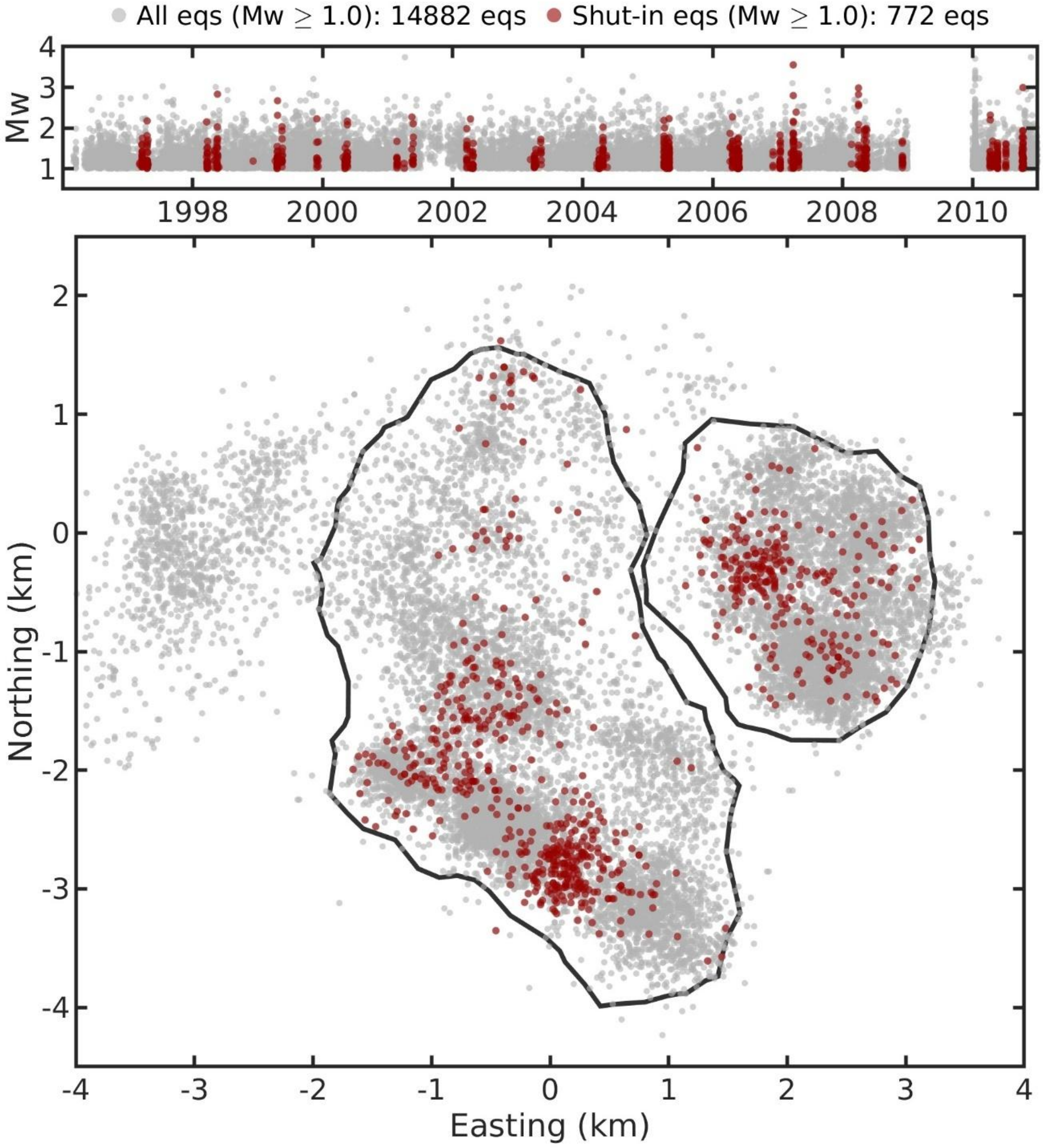

Figure S1. Timeline (top) and map (bottom) of shut-in seismicity at the Coso Geothermal Field (CGF) from Holmgren et al. (2025, *The Seismic Record*). Only earthquakes larger than moment magnitude (M_w) 1.0 (i.e., the magnitude of completeness) are shown. Shut-in events are shown in red. Map is created using MATLAB (version R2024b, https://www.mathworks.com) with subfield outlines extracted by clustering the earthquake catalog using DBSCAN (epsilon neighborhood = 260 m and minimum neighbors = 45).

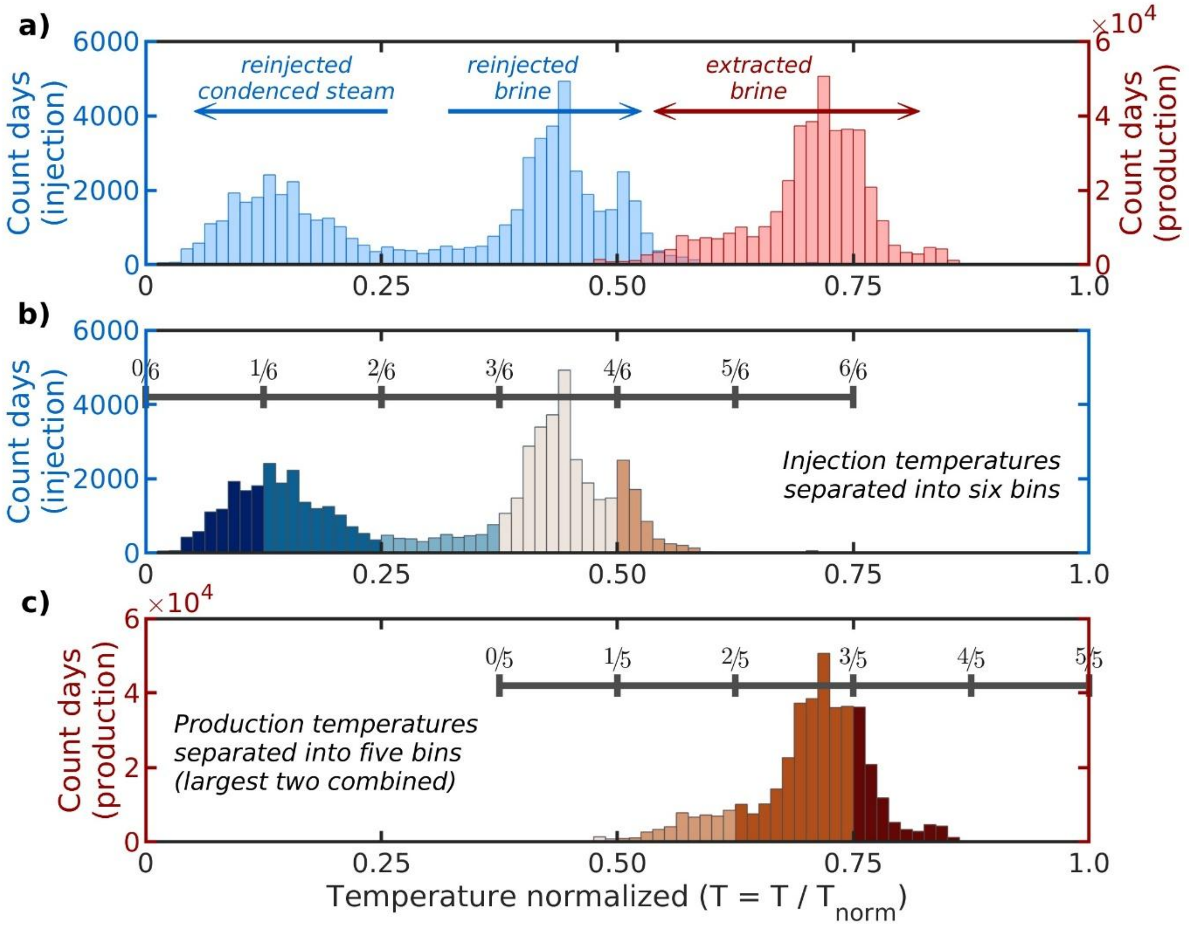

Figure S2. Normalization method used for injection and production data. (a) Temperature histograms for the full field's daily injection (blue) and production (red) data, showing the number of days each temperature bin was recorded between 1996 and 2010. All temperature data (both injection and production) is initially normalized by T_{norm}, where 0.0 = 0°C and 1.0 = T_{norm}. The bimodal distribution of the injection data shows the two endmembers colder condensed steam and hotter brine being reinjected. (b) Injection temperature data separated into six bins of equal temperature span. (c) Production temperature data separated into five bins of equal temperature span. The bins are used to separate the operational volume data based on temperature and the bin colors in (b) and (c) are the same as in Figures 3, 4, S6 and S9-S11. Note that the bin widths are the same for injection and production data, the production temperature data just covered a smaller temperature span in total. Furthermore, the two warmest bins in the production data have been combined because of the limited number of recordings in the 4/5-5/5 bin.

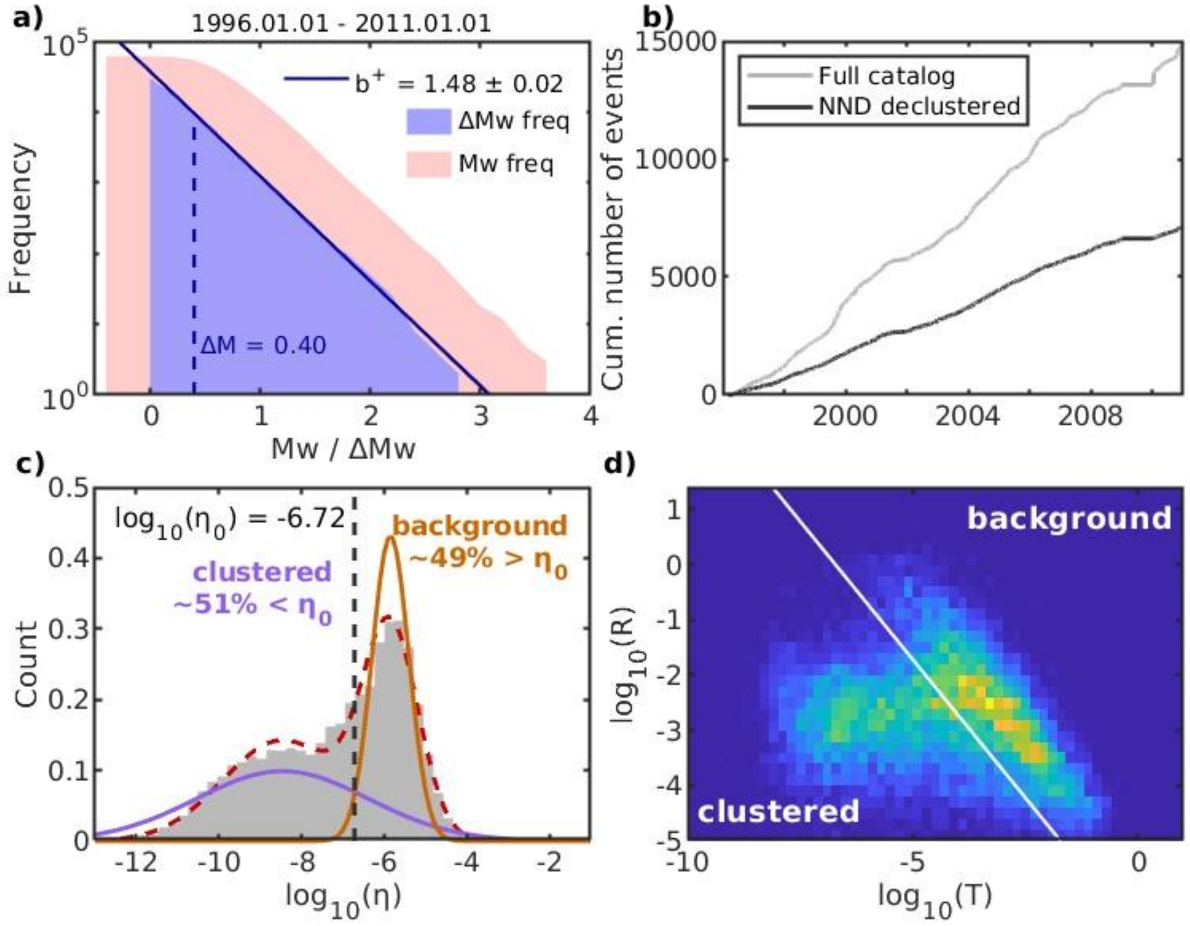

Figure S3. Nearest-neighbor distance (NND) declustering overview. (a) Moment magnitude (M_w) and ΔM_w frequency distributions for the CGF catalog, along with the b^+ estimate using a cut-off at ΔM_w = 0.4. (b) Cumulative number of events before and after NND declustering. (c) η histogram with the fitted Gaussian mixture model. (d) T-R histogram with $\log_{10}(\eta_0)$ shown as a white line.

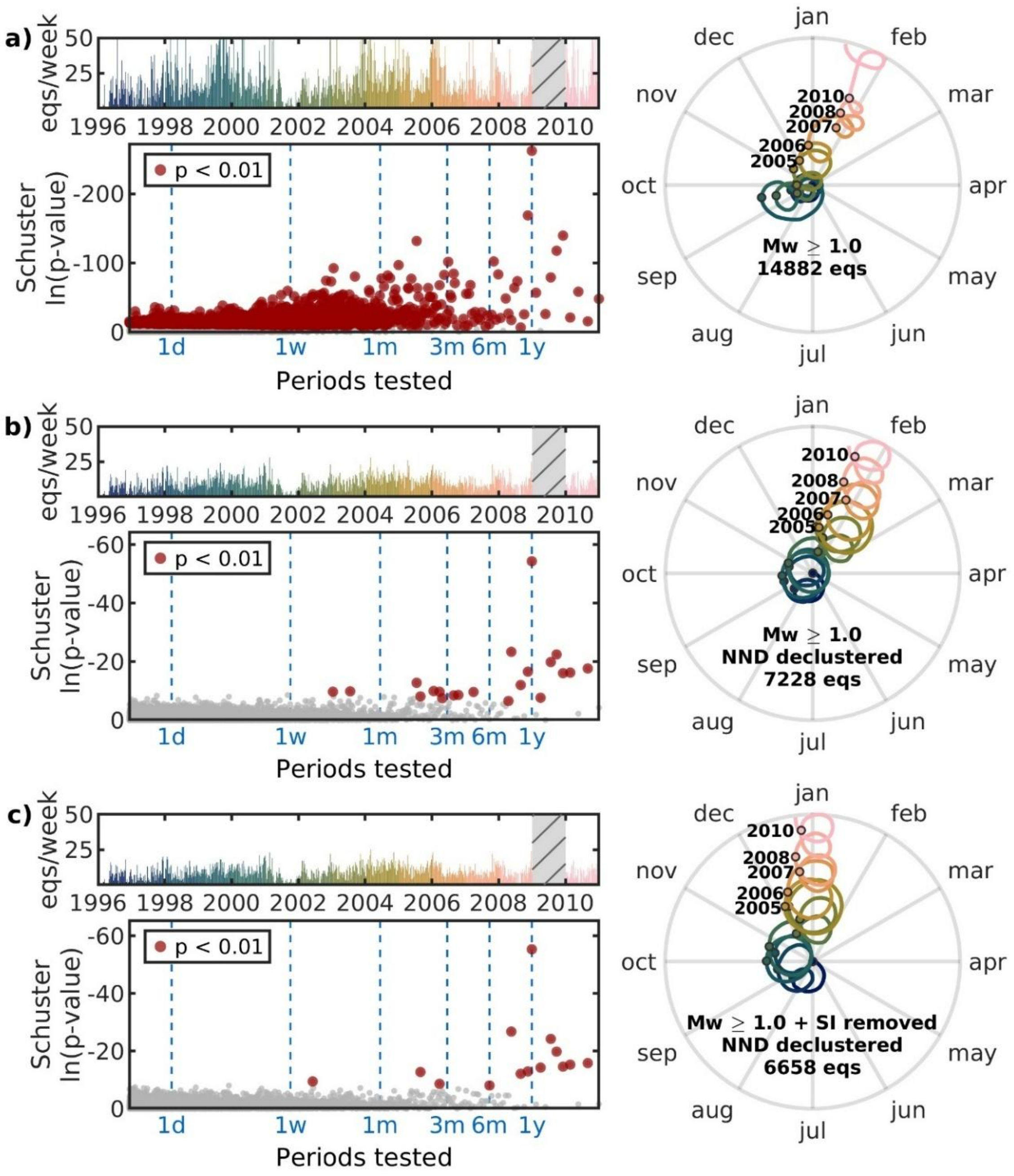

Figure S4. Periodicity analysis of the Coso Geothermal Field (CGF) catalog ($M_w \geq 1.0$) for (a) full catalog, (b) NND declustered catalog including shut-in (SI) events, and (c) SI events removed prior to NND declustering. For each catalog, plots similar to Fig. 2a-2c are shown: the upper left subplot shows the weekly earthquake rate, bottom left shows the Schuster spectrum, and right shows the polar walk (2D random walk). Panel (c) is identical to Fig. 2a-2c. The full earthquake catalog (a) shows that there is a significant presence of swarms and clustering (indicated by the many red dots in the Schuster diagram and the straight polar walk line in 2010), whereas the declustered versions have much fewer prominent peaks and only T = 1 year as the most significant. Furthermore, by removing the SI events, the polar walk tilts towards January, which is expected as CGF shut ins tend to occur in the springtime.

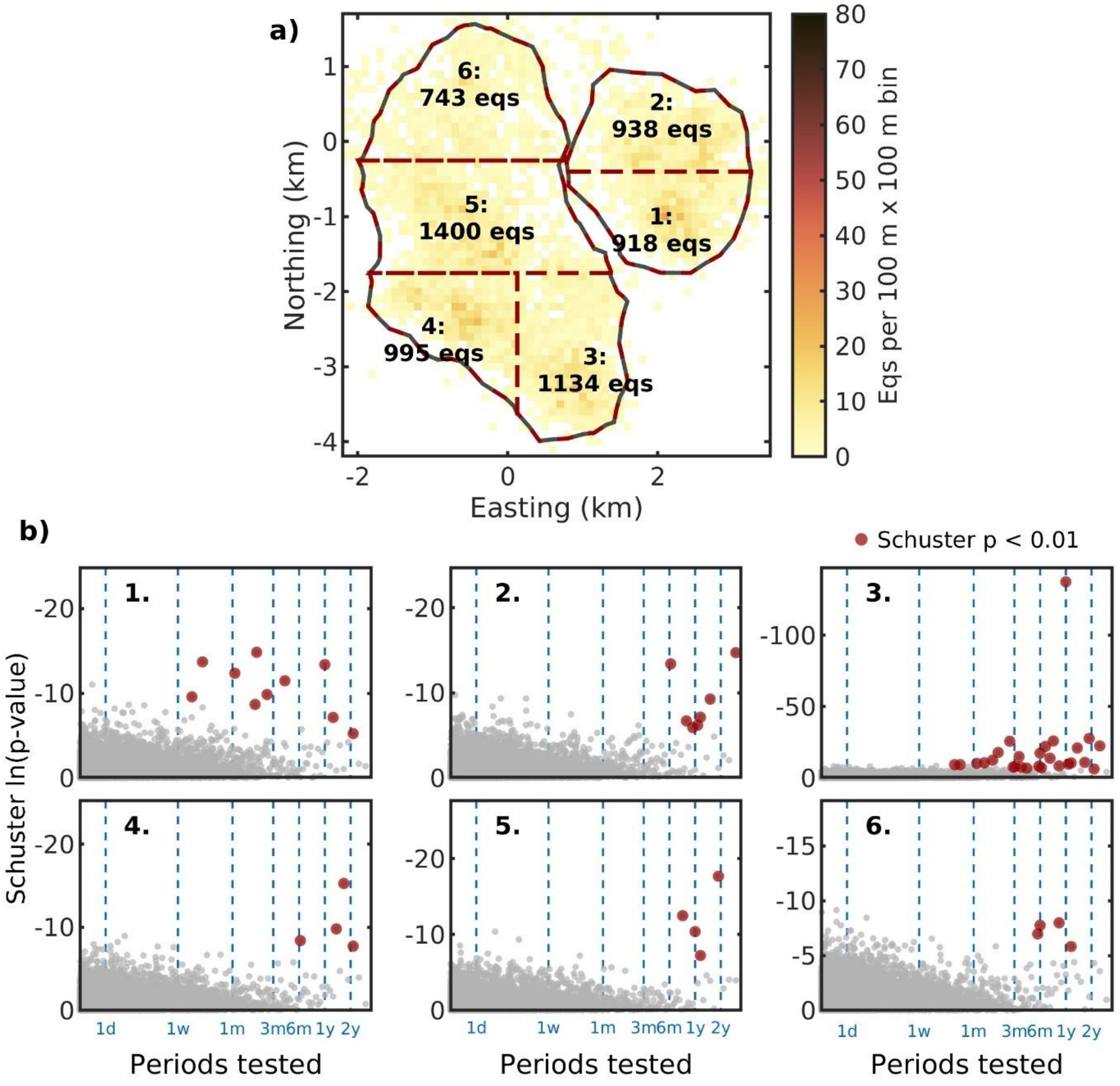

Figure S5. Spatial variation in Schuster spectrum for the Coso Geothermal Field (CGF). (a) Earthquake density map of the NND declustered catalog, with the CGF separated into six regions. (b) Schuster spectrum for each of the six regions in (a). Schuster p-value below 0.01 (99% confidence level) are highlighted in red. Region 3 exhibits a distinctly significant Schuster p-value at T = 1 year, whereas the remaining p-values below 0.01 are likely due to sudden bursts of seismicity with duration less than T, or the presence of not fully independent events clustering in time (such as swarms or aftershock sequences) despite the declustering process. Map is created using MATLAB (version R2024b, https://www.mathworks.com) with subfield outlines extracted by clustering the earthquake catalog using DBSCAN (epsilon neighborhood = 260 m and minimum neighbors = 45).

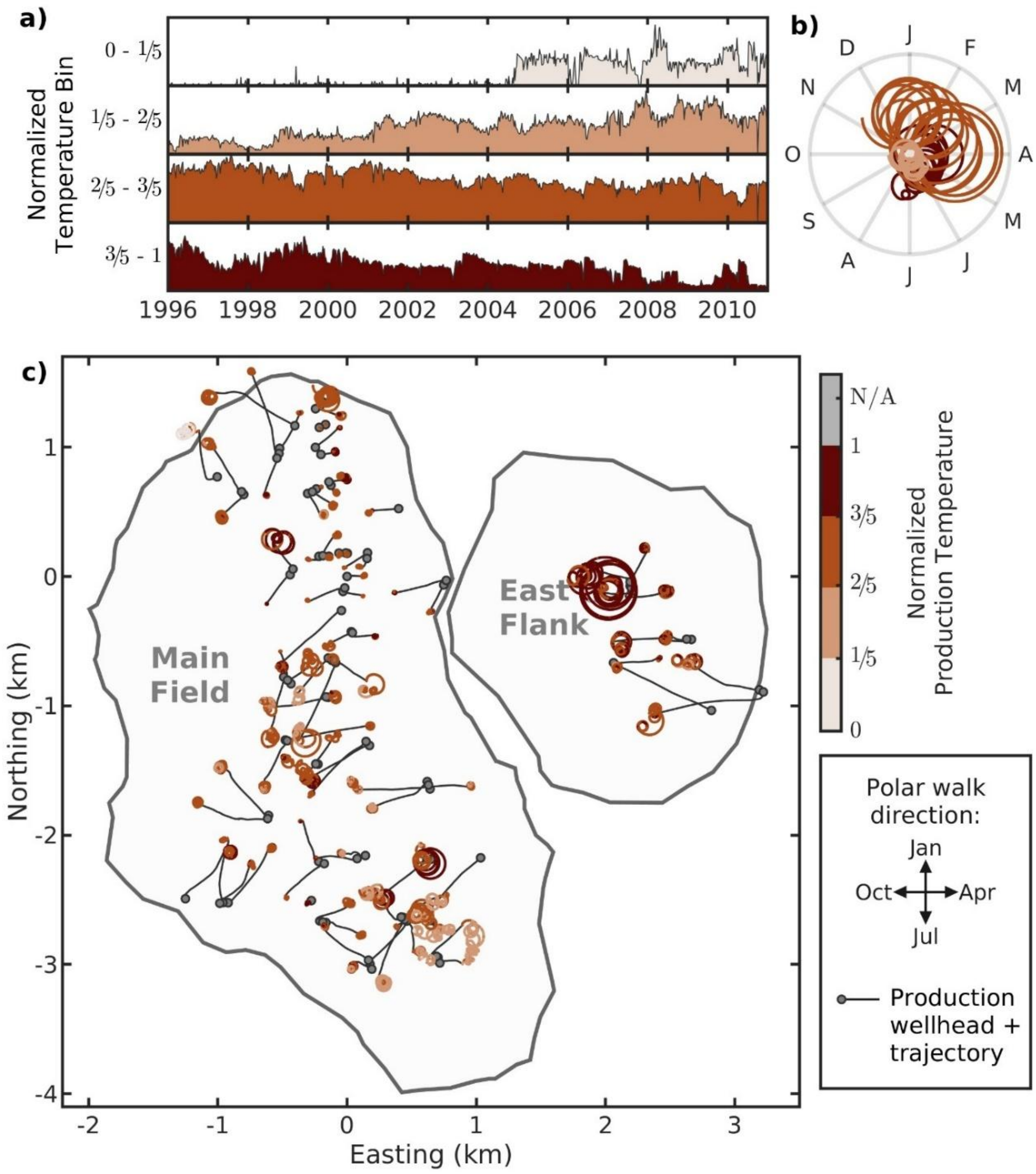

Figure S6. Same as Figure 3 but showing production wellbore data. The production temperature is linearly normalized and separated into five bins, where 0 is the coldest produced temperatures recorded and 1 is the warmest. Note, bins 3/5 and 4/5 have been combined as there was very little production in the 4/5–1 temperature bin during the study period. No clear $T = 1$ year periodicity is apparent for the production data, indicated by the circular polar walks in (b) and (c). The size of the polar walk circles corresponds to the total amount of volume being produced at that well, the larger the circle the more volume is extracted. See Figure S2 for a visualization of the temperature normalization. Map is created using MATLAB (version R2024b, https://www.mathworks.com) with subfield outlines extracted by clustering the earthquake catalog using DBSCAN (epsilon neighborhood = 260 m and minimum neighbors = 45).

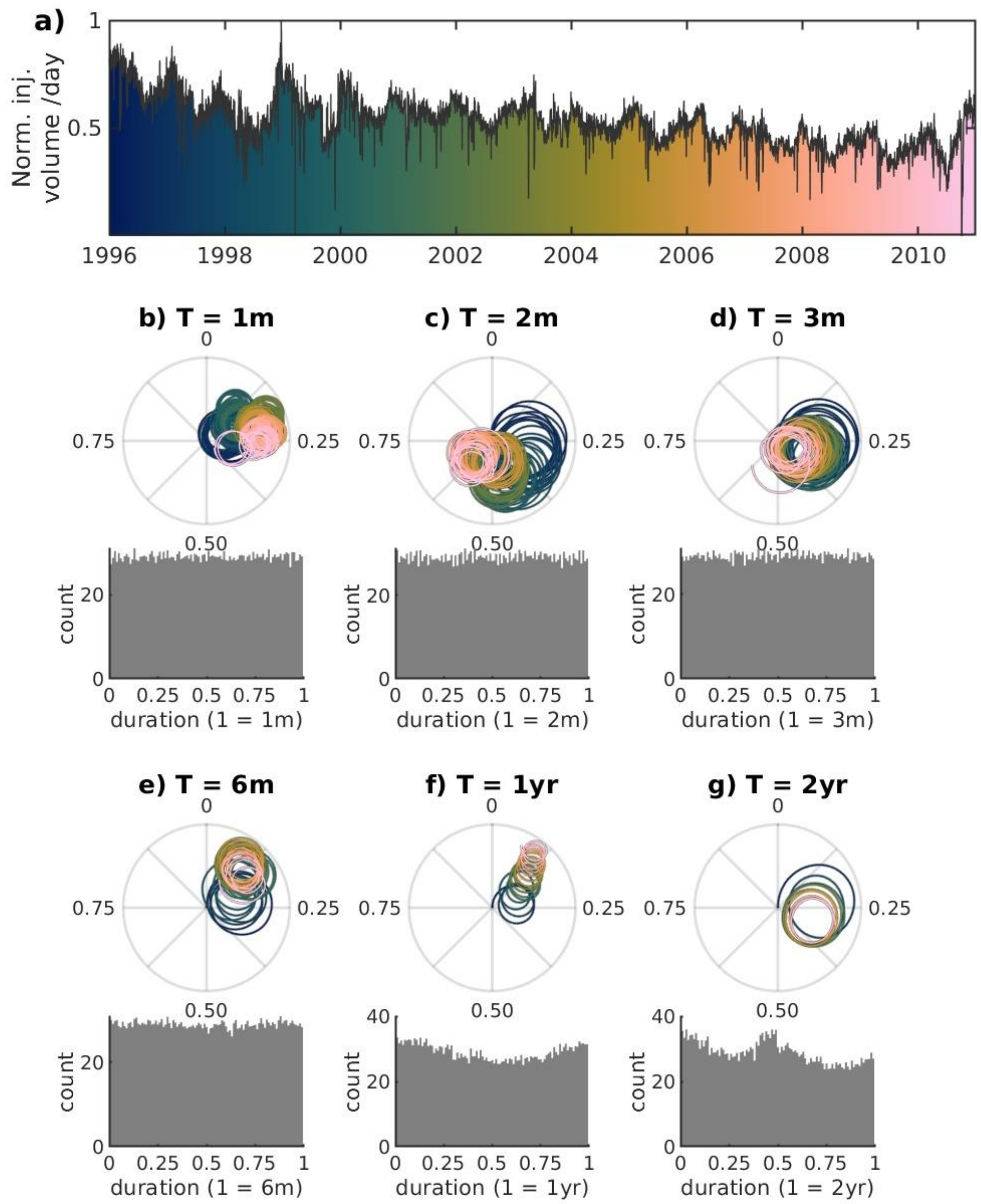

Figure S7. Periodicity investigation of the daily injection data throughout the study period 1996-2010. (a) Daily normalized injection volume, where each bar is colored by time stamp. Polar walks and corresponding histograms for six different temporal bins used to investigate periodicity trends: (b) 1 month, (c) 2 months, (d) 3 months, (e) 6 months, (f) 1 year, and (g) 2 years. For each temporal bin, the upper panel shows the polar walk representation of the data, colored by time stamp consistent with (a). Periodicity is indicated by polar walk circles moving successively away from the origin, which is only displayed by the 1-year bin (f). The lower panels provide the same information as histograms, where the data are binned according to the corresponding time window (e.g., for $T = 1$ month, values 0-1 represent the day of month). Again, only $T = 1$ year (f) displays non-uniform temporal trends. $T = 2$ years histogram (g) displays the annual trend again (as there is an extra bump at 0.5 = 1 year).

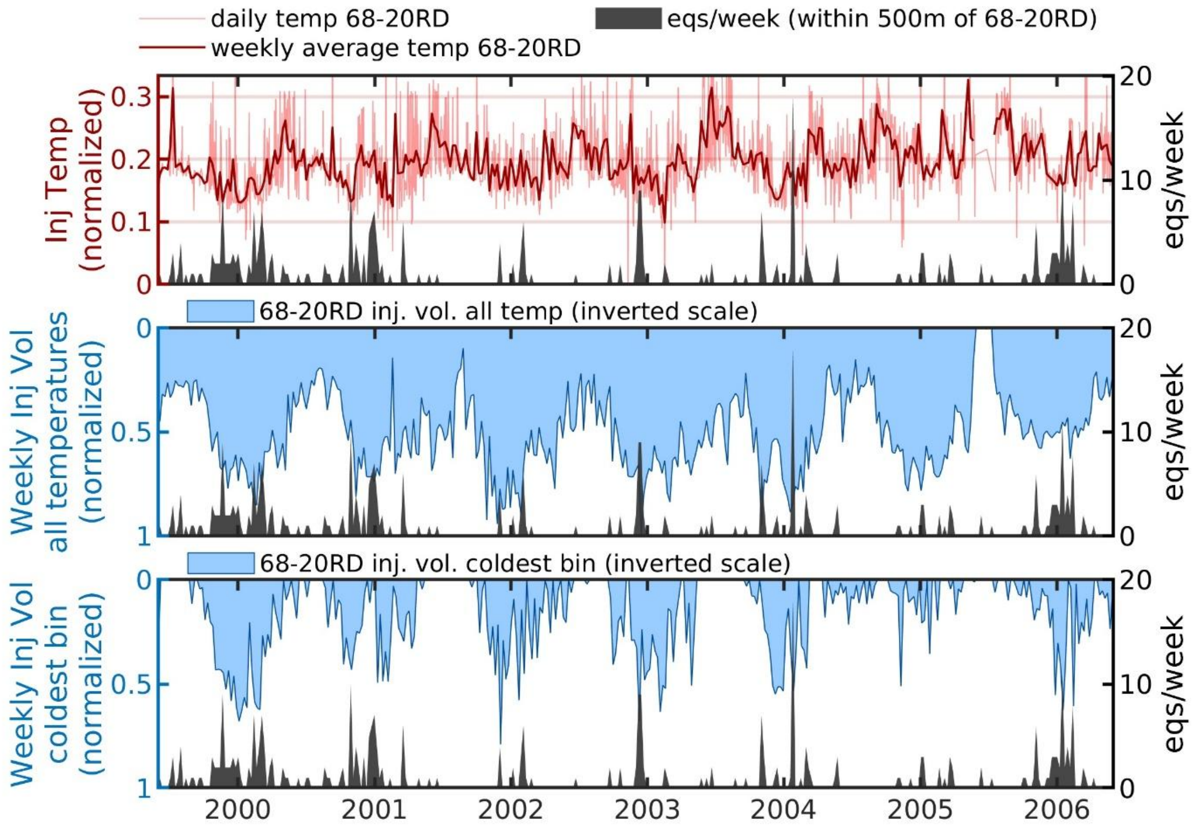

Figure S8. Zoom-in of injection temperature and volume for wellbore 68-20RD between 1999 and 2006 (peak periodicity years). Top panel shows the daily and weekly injection temperature (normalized following Figure S2b). Middle and bottom panels show the weekly injection volume for all temperatures (i.e., all injection data) and the coldest temperature bin, respectively. Note that these have been inverted in the panels. The black histogram in all three panels show the number of earthquakes per week ($M_w \geq 1$) within 500 m epicentral distance of wellbore 68-20RD.

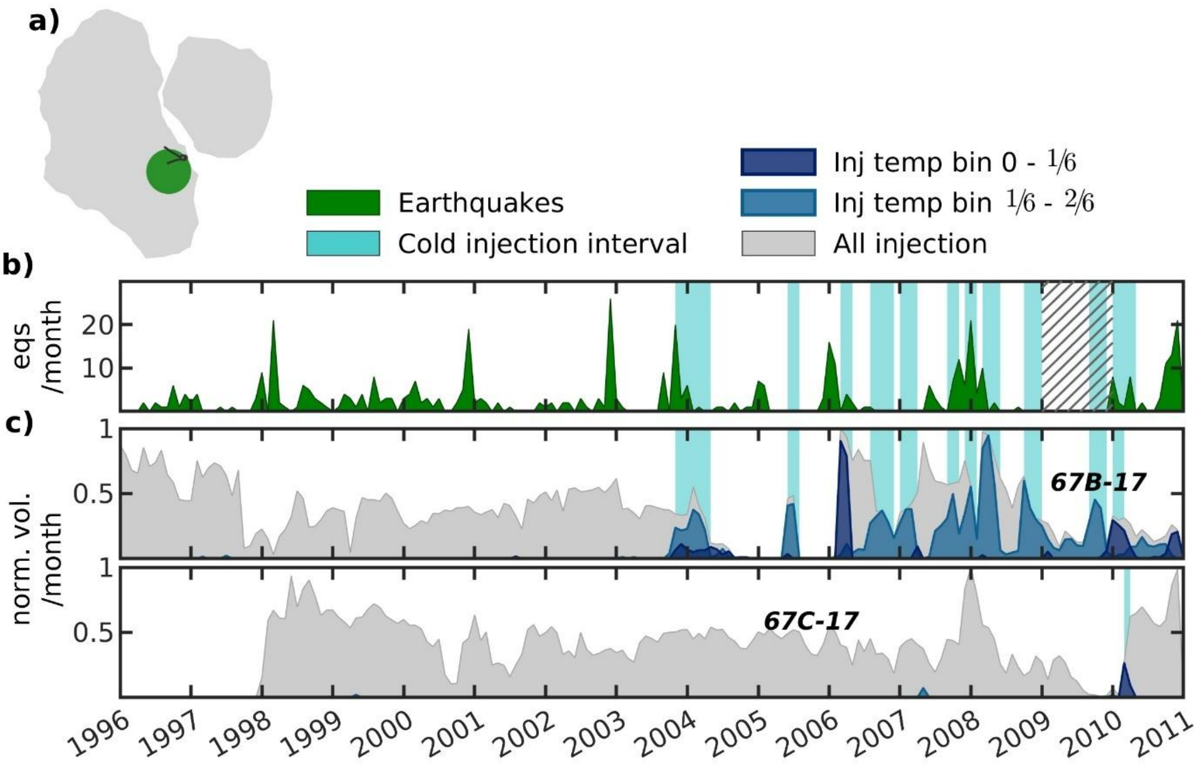

Figure S9. Similar to Figure 4 but showing the central-eastern Main Field region (shown in Figure 4a and 4b as orange region) by itself along with data from nearby injection wells 67B-17 and 67C-17. Light blue indicates cold injection intervals when the combined 0–1/6 and 1/6–2/6 temperature bins' volume exceeds 10% of the well's maximum monthly injection volume. Map is created using MATLAB (version R2024b, https://www.mathworks.com) with subfield outlines extracted by clustering the earthquake catalog using DBSCAN (epsilon neighborhood = 260 m and minimum neighbors = 45).

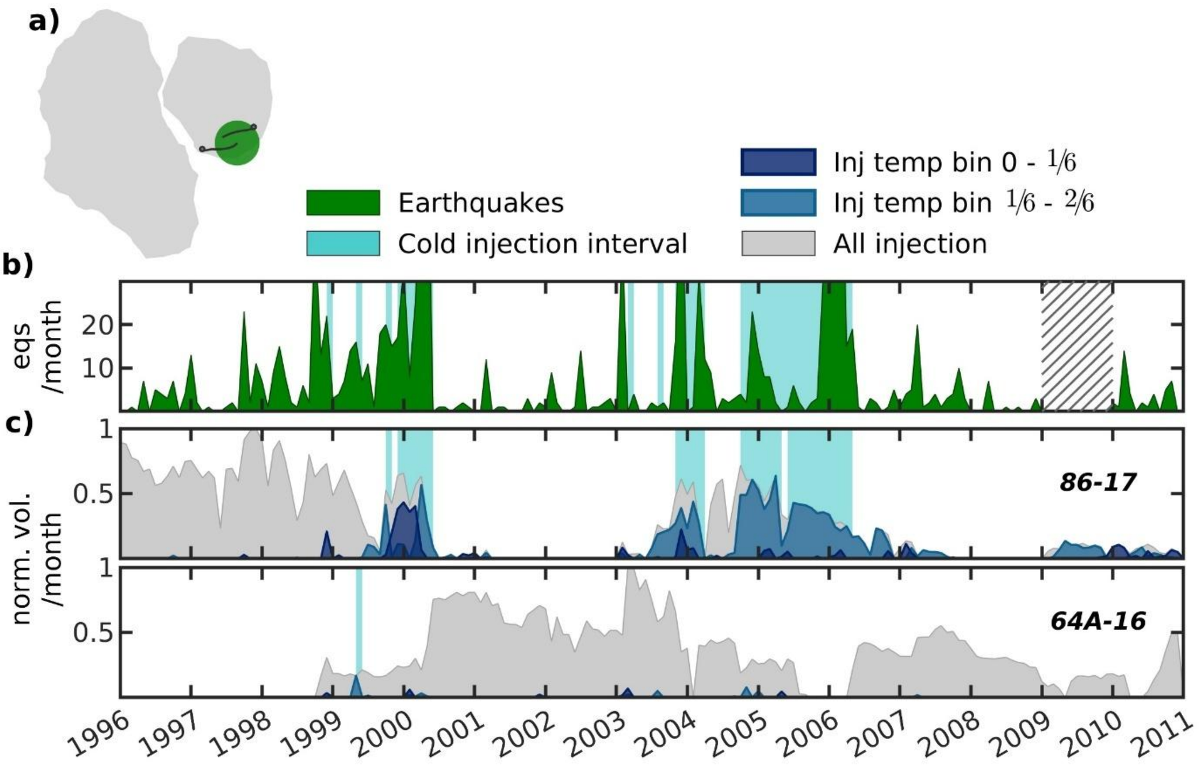

Figure S10. Similar to Figure 4 but showing seismicity and injection trends for the southern East Flank region with increased yearly periodicity trend and data from the nearby injection wells 86-17 and 64A-16. Light blue indicates cold injection intervals when the combined 0–1/6 and 1/6–2/6 temperature bins' volume exceeds 10% of the well's maximum monthly injection volume. Map is created using MATLAB (version R2024b, https://www.mathworks.com) with subfield outlines extracted by clustering the earthquake catalog using DBSCAN (epsilon neighborhood = 260 m and minimum neighbors = 45).

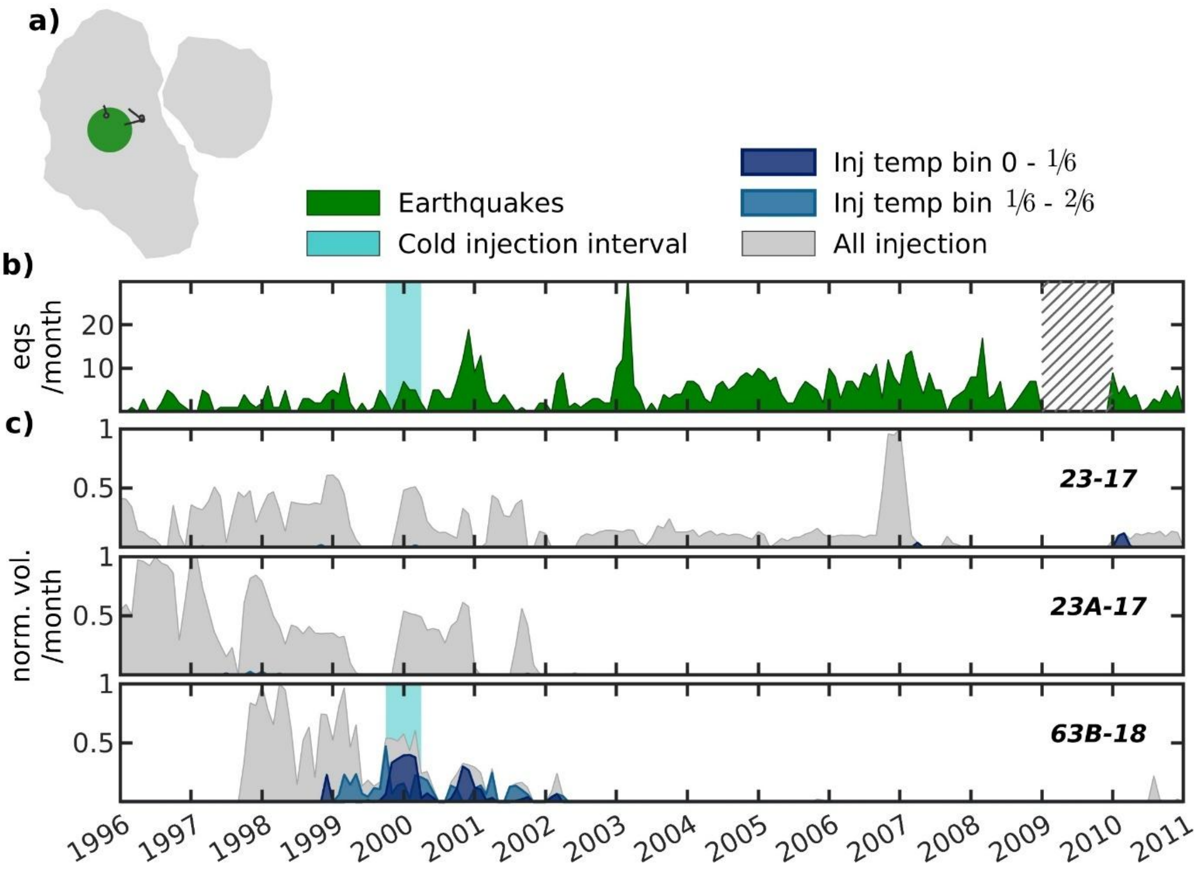

Figure S11. Similar to Figure 4 but showing the central Main Field region with increased yearly periodicity trend along with data from nearby injection wells 23-17, 23A-17, and 63B-18. Light blue indicates cold injection intervals when the combined 0–1/6 and 1/6–2/6 temperature bins' volume exceeds 10% of the well's maximum monthly injection volume. Map is created using MATLAB (version R2024b, https://www.mathworks.com) with subfield outlines extracted by clustering the earthquake catalog using DBSCAN (epsilon neighborhood = 260 m and minimum neighbors = 45).

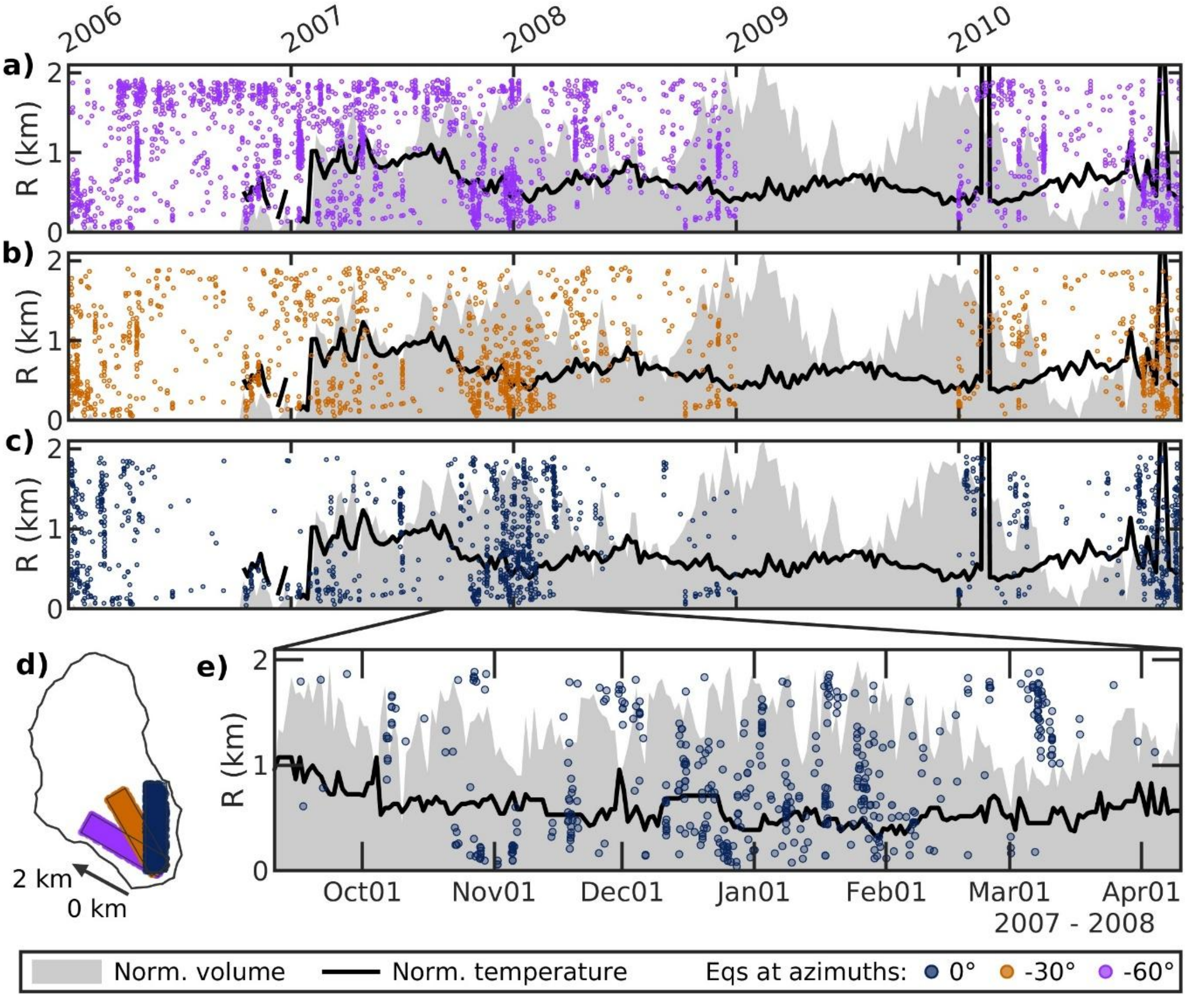

Figure S12. Similar to Figure 5 but showing injection volume and temperature for wellbore 68B-20RD between 2006-2011 during primarily condensed steam injection. (a)-(b) show weekly normalized injection volumes and temperatures in the background, whereas (e) shows daily normalized injection volumes and temperatures. (e) is a zoom-in of an increased seismicity period that coincides with a decrease in injection temperature rather than increase in mass injected. Map is created using MATLAB (version R2024b, https://www.mathworks.com) with subfield outline extracted by clustering the earthquake catalog using DBSCAN (epsilon neighborhood = 260 m and minimum neighbors = 45).

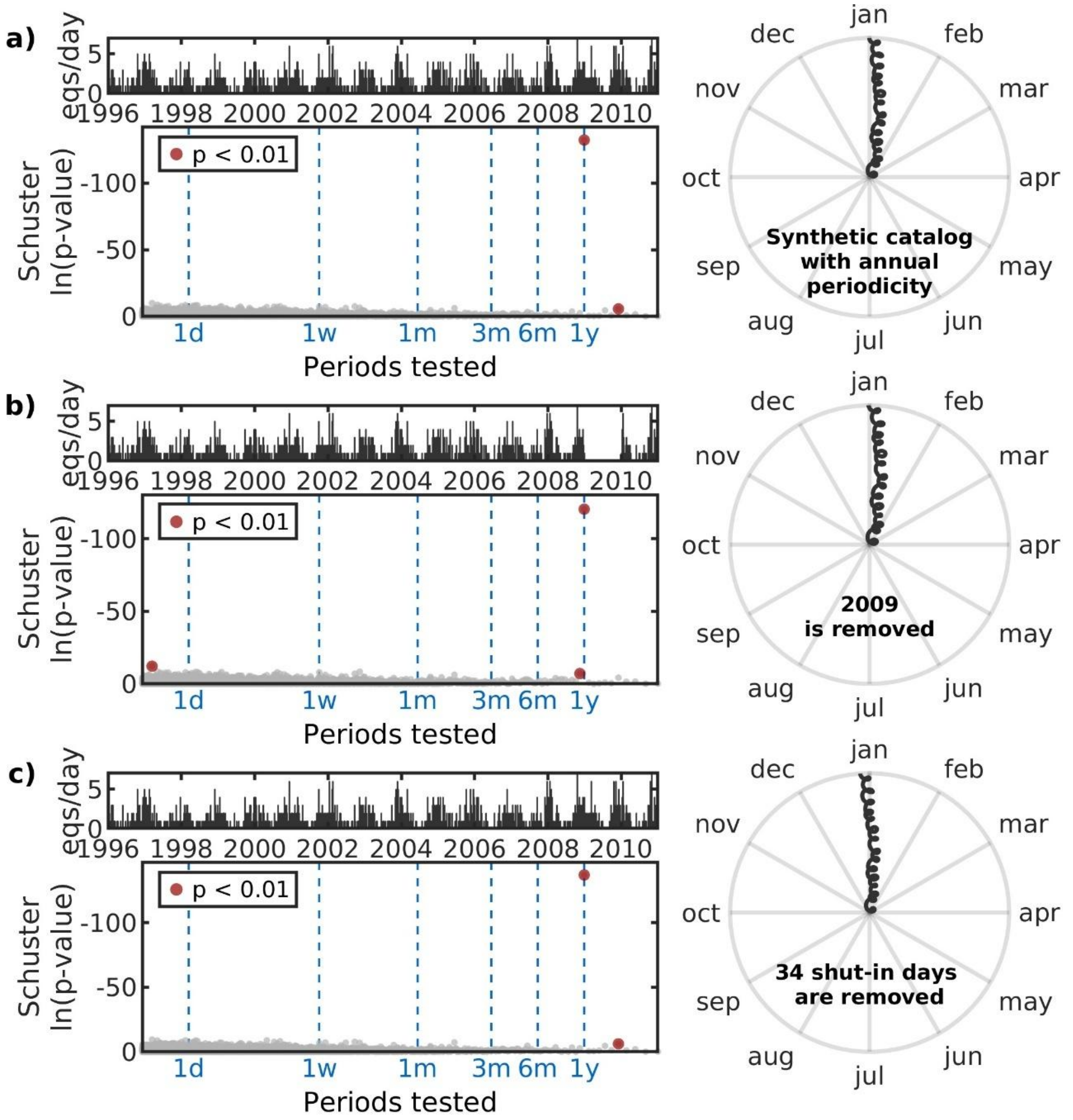

Figure S13. Examining how periodicity is affected by removing one full year or a few days each spring from a synthetic earthquake catalog with a strong annual periodicity between 1996 and 2010. (a) Timeline of the daily earthquake rate for the full synthetic catalog (top left), resultant Schuster spectrum (top left) and polar walk for period = 1 year (right). (b) and (c) show the same but when 2009 and the 34 shut-in days are removed, respectively. As can be seen, neither affects the periodicity results significantly.
